# Geomicrobiological Heterogeneity of Lithic Habitats in the Extreme Environment of Antarctic Nunataks: A Potential Early Mars Analog

**DOI:** 10.3389/fmicb.2021.670982

**Published:** 2021-07-02

**Authors:** Miguel Ángel Fernández-Martínez, Miriam García-Villadangos, Mercedes Moreno-Paz, Valentin Gangloff, Daniel Carrizo, Yolanda Blanco, Sergi González, Laura Sánchez-García, Olga Prieto-Ballesteros, Ianina Altshuler, Lyle G. Whyte, Victor Parro, Alberto G. Fairén

**Affiliations:** ^1^Centro de Astrobiología, CSIC-INTA, Madrid, Spain; ^2^Department of Natural Resource Sciences, Faculty of Agricultural and Environmental Sciences, McGill University, Sainte-Anne-de-Bellevue, QC, Canada; ^3^Antarctic Group, Agencia Estatal de Meteorología, Barcelona, Spain; ^4^Department of Astronomy, Cornell University, Ithaca, NY, United States

**Keywords:** polar microbiology, nunatak, environmental microbiology, terrestrial analogs of Martian habitats, astrobiology

## Abstract

Nunataks are permanent ice-free rocky peaks that project above ice caps in polar regions, thus being exposed to extreme climatic conditions throughout the year. They undergo extremely low temperatures and scarcity of liquid water in winter, while receiving high incident and reflected (albedo) UVA-B radiation in summer. Here, we investigate the geomicrobiology of the permanently exposed lithic substrates of nunataks from Livingston Island (South Shetlands, Antarctic Peninsula), with focus on prokaryotic community structure and their main metabolic traits. Contrarily to first hypothesis, an extensive sampling based on different gradients and multianalytical approaches demonstrated significant differences for most geomicrobiological parameters between the bedrock, soil, and loose rock substrates, which overlapped any other regional variation. *Brevibacillus* genus dominated on bedrock and soil substrates, while loose rocks contained a diverse microbial community, including Actinobacteria, Alphaproteobacteria and abundant Cyanobacteria inhabiting the milder and diverse microhabitats within. Archaea, a domain never described before in similar Antarctic environments, were also consistently found in the three substrates, but being more abundant and potentially more active in soils. Stable isotopic ratios of total carbon (δ ^13^C) and nitrogen (δ ^15^N), soluble anions concentrations, and the detection of proteins involved in key metabolisms *via* the Life Detector Chip (LDChip), suggest that microbial primary production has a pivotal role in nutrient cycling at these exposed areas with limited deposition of nutrients. Detection of stress-resistance proteins, such as molecular chaperons, suggests microbial molecular adaptation mechanisms to cope with these harsh conditions. Since early Mars may have encompassed analogous environmental conditions as the ones found in these Antarctic nunataks, our study also contributes to the understanding of the metabolic features and biomarker profiles of a potential Martian microbiota, as well as the use of LDChip in future life detection missions.

## Introduction

Only a small area on Antarctica is permanently ice-free, corresponding to approximately 49,500 km^2^ (0.35–0.40% of total surface (Bockheim et al., 2015; Convey et al., 2018). Half of these ice-free 49,500 km^2^ correspond to the Transantarctic Mountains, while other 20% are located along the Antarctic Peninsula region (Bockheim et al., 2015). Exposed terrains in most of the Antarctic ice-free regions mainly consist of sandy material with very low organic matter and microbial populations (Lambrechts et al., 2019). Therefore, they can be considered representatives of one of four different orders of soil, i.e. Gelisols, Entisols, Inceptisols and Histosols (Cowan et al., 2014; Bockheim et al., 2015; Garrido-Benavent et al., 2020). Particular formations within these exposed areas are the peaks from a mountain range that protrude above a surrounding ice cap or glacier, also known as nunataks (from Inuit *nunataq, lonely peak*). Nunataks usually present a small alteration of soils that even allows bedrock to be studied. Thus, they represent unique, mostly pristine environments that can be considered as ideal natural laboratories for polar studies. Although nunataks can be found in continental and peninsular (including archipelagos close to it) regions of Antarctica, they have received little attention from a biological perspective, with only a few studies analysing nunatak microbiology in the last decades [e.g., (Brinkmann et al., 2007; Yergeau et al., 2009), both studying Coal Nunatak in Alexander Island, 72°03' S 68°31' W, and (Almela et al., 2021), not focused but including samples from Clark Nunatak, Byers Peninsula, Livingston Island].

Within lithic environments, soils are characterized by having an enormous heterogeneity in geochemical parameters that leads to niche partitioning, including variations in vertical profiles in the scale of a few centimeters (Cary et al., 2010; Bottos et al., 2014; Bockheim et al., 2015; Almela et al., 2021). Consequently, these ecosystems usually contain high microbial diversity – discoveries of novel lineages are frequent – with reduced space for the different populations to develop, and a higher influence of stochastic events on the inhabiting communities (Bottos et al., 2014; Feeser et al., 2018). To date, microbial communities from exposed Antarctic soils have received moderate attention, despite their importance to ecosystem function (Cary et al., 2010; Bottos et al., 2014; Niederberger et al., 2015; Feeser et al., 2018; Lambrechts et al., 2019; Garrido-Benavent et al., 2020; Almela et al., 2021). These microbial communities are mainly composed of psychrotolerant organisms, with maximum growth rates at temperatures below 20°C. Thus, they have evolved metabolic adaptations, such as improved water uptake mechanisms, increased presence of exo-polysaccharides (EPS) as ‘cryoprotectant’, enhanced enzymatic efficiency, mechanisms to cope with protein denaturation and misfolding, as well as a remarkable functional resilience (Bakermans et al., 2012; Doyle et al., 2012; Chong et al., 2015). They also demonstrate a high proportion of unsaturated lipids and pigments to increase membrane fluidity and UV radiation resistance, respectively. Though many detected microorganisms remain uncultivated, the principal described bacterial taxa include Actinobacteria and Proteobacteria as the major phyla (Cary et al., 2010; Lambrechts et al., 2019; Garrido-Benavent et al., 2020). The Archaea domain has been much less studied in these ecosystems, showing a restricted distribution and lower concentration of cells, with no members isolated from soil ice-free areas other than geothermally heated ones (Niederberger et al., 2012; Amenábar et al., 2013; Bendia et al., 2018; Lambrechts et al., 2019). Described Antarctic soil archaeal communities mainly included members within Euryarchaeota, Thaumarchaeota and Crenarchaeota phyla (Bates et al., 2011; Richter et al., 2014; Pessi et al., 2015; Zhang et al., 2020).

Several Antarctic microbial taxa have developed strategies to colonize rocks as their primary niche (i.e., ‘lithobionts’), following an avoidance strategy since rocks harbor a more stable environment inside (De Los Ríos et al., 2014; Yung et al., 2014; Coleine et al., 2020). Endolithic microorganisms can thus colonize different spaces within rocks: chasmoendoliths appear on cracks and fissures in contrast to cryptoendoliths, which grow on pores formed after weathering of rocks (Pointing et al., 2009; Cowan, 2014; De Los Ríos et al., 2014; Archer et al., 2016). This endolithic community establishment requires the formation of complex biofilms within the cracks and pores and sometimes lead to a layered pattern of colonization, following location of radiation-protecting pigments or even different microbial communities (Friedmann, 1982; Pointing and Belnap, 2012; De Los Ríos et al., 2014; Yung et al., 2014; Archer et al., 2016; Crits-Christoph et al., 2016; Wierzchos et al., 2018). Specifically, rocks offer a shelter against surrounding climatic harshness and UV radiation, acting as a reservoir of water and a warmer environment (up to 5–15°C above air temperature in specific thermal flux areas). However, rocks, and especially bedrock, are highly oligotrophic environments (Kirby et al., 2012; Amarelle et al., 2019). Consequently, it has been suggested that the principal carbon and nitrogen inputs for nutrient cycling in endolithic environments come from photoautotrophic and diazotrophic microorganisms (Cowan et al., 2014; Amarelle et al., 2019; Garrido-Benavent et al., 2020). Hence, lithobiontic communities in Antarctica are primarily composed of the Cyanobacteria and Proteobacteria phyla, but with a high incidence of fungi and lichens (de la Torre et al., 2003; Cary et al., 2010; De Los Ríos et al., 2014; Benavent-González et al., 2018; Amarelle et al., 2019; Garrido-Benavent et al., 2020), while Archaea have been recently described as absent from rock-inhabiting communities in such extreme environments (Crits-Christoph et al., 2016). While many studies employed microscopy to describe Antarctic microbial lithic communities [reviewed in Amarelle et al. (2019)], amplicon sequencing and/or microarray approaches have been less employed (e.g., Garrido-Benavent et al., 2020), in contrast to the studies of lithobiontic communities from other extreme environments, such as the Atacama Desert (Wierzchos et al., 2018). Additionally, very few studies on extreme environments have focused on bedrock samples, which constitute an uncharted habitat for microbial communities that deserve further investigations.

In this work, we compare the different sources of variations of the previously uncharacterized geomicrobiology of permanently ice-free regions in the Hurd Peninsula, Livingston Island, in the Antarctic Peninsula area. Due to environmental differences expected within the Hurd Peninsula – especially in terms of UV radiation following North vs. South orientation of the terrains – we hypothesized that the variability across sampling sites overlaps low-scale variations that could be present within the different lithic substrates (bedrock, soil, or loose exposed rocks) – inhabited by the abovementioned microbial communities. To contrast this hypothesis and to identify the main geochemical and climatological drivers of the microbial life in these niches, we follow a dedicated sampling approach to study the influence of regional climatic conditions *vs*. low-scale gradients on microbial composition and metabolic traits. Samples were analyzed by a comprehensive set of geochemical, microscopical, and molecular biology techniques, including on site analysis with an in-house developed antibody microarray chip (Life Detector Chip-LDChip; Parro et al., 2011). Simultaneously, we were able to assess the potential of the Antarctic nunataks as terrestrial Mars paleoenvironments analogs, in comparison with other Antarctic areas (e.g., Dry Valleys) which are mostly considered analogs to current Martian environments. Mars today is a hyperarid barren desert, but during the first billion years of its geological history a hydrological cycle was active on a then milder, water-enriched planet, with saline and acidic liquid solutions stable even at the prevailing sub-zero temperatures (Fairén, 2010; Fairen et al., 2010). This approach with the fact that LDChip is the core sensing of Signs of Life Detector (SOLID), a TRL 6 instrument developed for *in situ* life detection on Mars (Parro et al., 2011), allows us to also frame this study within an Astrobiology context.

## Materials and Methods

### Site Description

#### Geology

**Figure 1:**
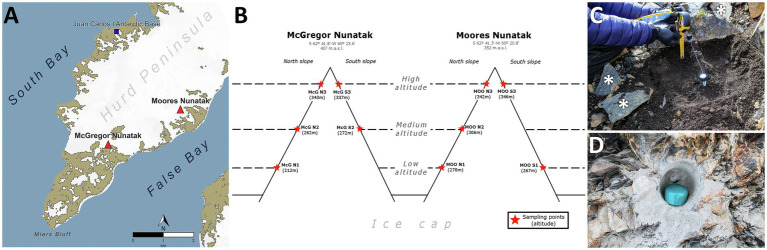
Study and sampling location. **(A)** Location of sampled nunataks and Juan Carlos I Spanish Antarctic Base at Hurd Peninsula, Livingston Island (South Shetlands). **(B)** Sampling points distribution along Northern and Southern slopes at both McGregor and Moores nunataks. **(C)** Example of manual sampling of both soils and individual loose rocks (asterisk) at every sampling point. **(D)** Example of drilling hole for bedrock substrate sampling at every sampling point.

Livingston Island is the second largest (798 km^2^) of the South Shetlands Islands, an archipelago closely located to the Antarctic Peninsula. Hurd Peninsula, located in the southern half of Livingston Island, comprises about 27 km^2^, from South Bay in the northwest to False Bay in the southeast (Figure 1A). The interior of the peninsula is mainly covered by the temperate ice cap of Johnsons’ tidewater glacier, flowing northwestwards, and Hurd Glacier, which presents various land-terminating tongues flowing southwestwards (Molina et al., 2007; Rodríguez Cielos et al., 2016). Two main lithological sequences in the exposed areas of the peninsula emerge from the ice cap as nunataks and surround the milder terrestrial ends of Hurd Glacier (Garrido-Benavent et al., 2020). Mainly exposed to the north there is an overlying stratum of porous volcanic rocks belonging to South Shetlands Islands magmatic arc (Mount Bowles Formation); on their behalf, mostly exposed at the southeastern end of the peninsula, there are mainly conglomerates, sandstones, and mudstones from the Miers Bluff Formation, occupying a low stratigraphic position (Smellie et al., 1995). This Miers Bluff Formation has been characterized as very heterogeneous, showing a low-grade metamorphic turbidite sequence, as well as alternating quartzite – shales in layers, and several locations presenting numerous dykes; breccias are also present in the youngest parts of the formation (Smellie et al., 1995; Pimpirev et al., 2000; Zheng et al., 2003; Ferreira et al., 2017).

#### Climate

The Antarctic Peninsula and surrounding islands are characterized by a cold climate, nonetheless wetter than the Antarctic continent. This area exhibits a large interannual variability partially determined by El Niño-Southern Oscillation (ENSO) and the southern annual mode (SAM; Gonzalez and Vasallo, 2020). Specifically, Livingston Island is subjected to different synoptic patterns: the most frequent one transports warm and moist air from the southeast Pacific; least frequent one transports cold and dry air from the Antarctic continent on the south. This situation results in a dense cloud cover for most of the time (Spanish Meteorological Service, AEMET, personal communication; Gonzalez et al., 2018).

Specific meteorological data were obtained from the Antarctic Spanish Station Juan Carlos I (62.66°S, 60.39°W, 12 m.a.s.l.) and Hurd Glacier (62.69°S, 60.41°W, 93 m.a.s.l.) automatic weather stations (García and López, 2015). Data were retrieved from the quality-controlled Spanish Meteorological Service (AEMET) repository, with monthly averages obtained by averaging daily 10-min values for those months when at least 80% of data were available. A linear regression analysis was conducted on the Juan Carlos I and Hurd Glacier data to test the correlation of both datasets for the better interpretation of the macroclimatic conditions concerning nunataks slopes and the inhabiting microbial communities.

### Sampling

Samples were collected in January 2018 at McGregor and Moores Peaks, two nunataks located 2 km away, in an area close to the southeastern end of the Hurd Peninsula, on Livingston Island, Antarctica (Figure 1A Supporting Information Figure S1). Both nunataks were located within the Miers Bluff Formation, showing mainly turbidites as dominant bedrock, with outcrops of shales, especially at the McGregor nunatak, and breccias at the Moores nunatak. Sampling strategy was intended to describe four levels of variations for physicochemical parameters under the same environment/climatological setting (i.e., altitude, north/south orientation, nunatak location, and substrate, as depicted in Figure 1B). Briefly, wind flow speed and cold are expected to be higher with increasing altitude and insolation with northern orientation. Similarly, some differences were expected for physicochemical parameters between nunataks, due to their different location and distance from the sea. Therefore, sampling sites were established at three different altitudes along northern and southern slopes of both nunataks (Figure 1B); only the lowest and the medium altitude points at the south slopes of, respectively, McGregor and Moores nunataks were not safely accessible to collect the samples (lack of symbol in Figure 1B). Additionally, at each site, we collected samples from the three reachable lithic substrates in the area: (1) composite soil, (2) bedrock, and (3) loose rocks. Hereafter, samples are named in text with a code showing the substrate where they were obtained (‘SL’ for soil, ‘B’ for bedrock, and ‘R’ for loose rock samples) and four parameters relative to sampling point: (1) nunatak (‘McG’ or ‘MOO’), (2) slope (‘N’ or ‘S’), (3) altitude (1 = low altitude, 2 = medium altitude, or 3 = high altitude), and (4) – only for soil samples – ‘-SF’ standing for surface and ‘-10’ for 10-cm depth. As an example, ‘SL-McG-N1-SF’ represents a soil sample obtained from the McGregor nunatak at the lowest altitude of the northern slope and from the soil surface.

Sampling was carried out with autoclave-sterile tools (shovel, spatula, chisel, and hammer, 70° ethanol sterilized again after each collection of substrates) as follows: (1) soil samples were collected (*c*. 500 g) within a *c*. 25-cm-radius circumference established at areas devoid of any snow cover and with a dry surface (Figure 1C); after removing the uppermost soil layer (*c*. <1 cm) to avoid collecting material recently coming from higher altitude close sites, two independent samples were obtained, one from the remaining top layer (‘-SF’) and another from the 10-cm-depth layer (‘-10’). To avoid cross contamination from above soils, all the material between ‘SF’ and ‘10’ depths was aseptically discarded and 70° ethanol sterile tools were employed to sample this lowest depth. (2) Bedrock samples were collected (*c*. 100 g) after drilling with an autoclave- sterile head until a depth of *c*. 5 cm (Figure 1D); bedrock surface (*c*. <0.5 cm) was removed and the resulting surface, as well as the drill head, was sterilized with 70° ethanol prior to the in-depth drilling. (3) Loose rock samples were collected within *c*. 2-m-diameter circumference around the place where bedrock samples were obtained (Figure 1C, asterisk), avoiding those showing an obvious external colonization (i.e., lichens). All human manipulation was carried out wearing nitrile gloves, 70° ethanol sterilized several times during the sampling. All samples were placed in sterile Whirl-Pak^®^ bags and frozen at −80°C at the Juan Carlos I Antarctic Station facilities for storage until shipping and/or processing at the laboratory. Once in the laboratory and as a step prior to the loose rocks analysis, several different fragments (upper surface, interior, and soil-facing surface) were obtained with a chisel. All rock fragments were then crushed and grounded together – trying to capture epilithic, chasmoendolithic, cryptoendolithic, and hypolithic microbial communities for further analysis – using a sterile custom-designed steel pestle and mortar until a homogenized powder was obtained. Before each use, chisel, pestle, and mortar were sterilized with sodium hypochlorite (90% NaOCl – H_2_O), 70° ethanol, and UV-C radiation (30 mV, 5 min) using a GS gene linker UV chamber (Bio-Rad Laboratories, Hercules, CA, United States).

### Mineralogical, Petrological, and Geochemical Analyses

#### X-Ray Diffraction

Powder X-Ray Diffraction (hereafter, XRD) was performed on all lithic samples using a Seifert 3003 TT with Cu Kα radiation (*λ* = 1.542 Å). The X-ray generator acceleration voltage was 40 kV, with a filament emission of 40 mA. A step size of 0.1° (2θ) and a count time of 2 s were used to scan the samples with a range of measurement between 5° (2θ) and 60° (2θ).

#### Petrographic Characterization Through Thin Section Microscopy

Polished thin sections (20 × 30 mm, thickness 30 μm) of the rock samples were made by Geonatura INC. laboratory (Madrid, Spain). They were examined under polarizing/reflecting light with a Nikon Eclipse E600 POL petrographic microscope with lens of x5, x20, and x50 to identify textures, mineral compositions, and rock types. Pictures were obtained with an integrated Nikon DS-Fi2 camera.

#### Inductively Coupled Plasma-Mass Spectrometry

To carry out Inductively Coupled Plasma-Mass Spectrometry (hereafter, ICP-MS), all samples were dried in an oven at 40°C and milled down to a <70 μm size using an agate mortar. They were then stored in sterilized polypropylene (PP) vial switch caps for subsequent analysis. An amount of 200–300 mg of powdered samples were digested using a closed vessel-digestion microwave (Ethos Touch Control, Milestone Srl.) equipped with a high temperature and pressure rotor (1,500 psi and 300°C). Variable concentrations of HNO_3_, HCl, HF, and H_2_O_2_ (Suprapur^®^ grade, Merck KGaA) were employed depending on XRD results. Resultant solutions were brought to a final volume of 100 ml with deionized water in a Polymethylpentene (PMP) volumetric flask. Semiquantitative analysis was subsequently performed on every sample with a NexION 2000 ICP-MS spectrometer (PerkinElmer Inc.) – employing 47 elements as external standard and a ‘blank’ digestion sample to determine concentration levels prior to quantification – following standard laboratory protocols (Supporting Information Figure S1).

#### Ion Chromatography and pH

Ion chromatography (hereafter IC) was carried out in triplicate to test the presence of a group of soluble, inorganic, and organic anions as previously described (Fernandez-Martinez et al., 2019). Briefly, 2 g of sample was dissolved in 20 ml of distilled water with agitation for 2 days. Samples were then filtered through a 0.22 μm PTFE filter previously to be loaded into the chromatographer. Only those sample values that fit within calibration ranges were considered. The pH of soil samples was determined in triplicate by using a soil pH-meter directly on samples, while pH on loose rock and bedrock samples was measured in a suspension of substrate (substrate: Milli-Q water, 1:2.5) once in the laboratory.

#### Bulk Isotope Analysis

The stable isotopic composition of total organic carbon (δ^13^C) and total nitrogen (δ^15^N) in soil, bedrock, and loose exposed rock samples was measured in triplicates by isotope ratio mass spectrometry (IRMS). A MAT 253 (Thermo Fisher Scientific) IRMS equipment was used, following the analytical methods of the US Geological Survey (Révész et al., 2012). About 0.5 g of dried sample was grounded and homogenized using autoclave-sterile mortar and pestle, and carbonates were removed with HCl (3 M). After 24 h of equilibration, pH was adjusted to neutral values with ultrapure water, and the sample then dried in an oven (50°C) until stable weight (~48 h). The δ^13^C and δ^15^N ratios were reported in the standard per mil notation (‰) using three certified standards (USGS41, IAEA-600, and USGS40), with an analytical precision of 0.1‰. The elemental content of both TOC and TN was measured as percentage of dry weight (%) during stable isotope measurements, using Flash HT Elemental Analyzer (Thermo Fisher Scientific).

### DNA Extraction

Genomic DNA from soil and bedrock samples was independently extracted within 24 h after sampling using the EZNA soil DNA Kit (Omega Bio-Tek, Inc., Norcross, GA, United States) at Juan Carlos I Antarctic Base facilities, after homogenizing and mixing sampled material. DNA from loose rock samples was extracted in CAB’s laboratory (Madrid, Spain) using DNeasy PowerSoil Kit (Mo Bio Laboratories, Inc., Carlsbad, CA, United States), since it yielded a much higher DNA concentration for these low-biomass samples in comparison with EZNA kit. This rock sample DNA extraction was carried out with slight variations to standard procedures in order to maximize extracted DNA content and microbial diversity: for a single sample, four different extractions were carried out, each of them consisting on four subsamples (i.e., a total of 16 DNA *sub-extractions* per rock sample) subsequently eluted in a single volume of 100 μl at the final protocol step. Thus, we obtained *c*. 400 μl of extract (100 μl equimolarly pooled for each of the four extractions) per rock sample (Supporting Information Figure S2). Prior to all genomic DNA extractions, kit reagents and reaction tubes were sterilized with UV-C radiation (30 mV, 5 min). Three control extraction samples (one per substrate) were carried out to look for possible resistant kit reagent contamination, following all steps from both kits but with no sample included.

### 16S rRNA Gene Sequencing and Data Analysis

Bacterial and archaeal communities from soil, bedrock, and loose rocks were identified by the construction of paired-end amplicon libraries employing Illumina MiSeq sequencing technology (Illumina Inc., San Diego, CA, United States). The primer pair 341-F/805-R (Herlemann et al., 2011) was employed for bacterial 16S rRNA V3-V4 gene region amplification. Likewise, the primer pair Arch1F/Arch1R (Cruaud et al., 2014) was used to amplify archaeal 16S rRNA V2-V3 region. Both bacterial and archaeal PCRs, library preparation and sequencing processes were carried out at the NGS-Genomic Service of the Fundación Parque Científico de Madrid (FPCM) according to their protocols (Supporting Information Figure S2). Control samples from both DNA extraction and PCR amplification did not yield any positive results either by gel electrophoresis or by DNA concentration quantifications with Picogreen or Qubit techniques, so they were not included in the sequencing process. Raw sequence data were deposited at the NCBI Sequence Read Archive (SRA),^1^ under BioProject ID PRJNA723417.

Raw sequences were processed in MOTHUR software v.1.43.0 (Schloss et al., 2009), using a custom script based upon MiSeq SOP (Kozich et al., 2013), as it has been employed before by our group in Sanchez-Garcia et al., 2018, Azua-Bustos et al., 2019, Lezcano et al., 2019, Sanchez-Garcia et al., 2020b; further explained in Supporting Information Figure S3. To identify putative contaminant sequences remaining within the samples that were not detected with the negative control approach, ‘decontam’ R package (Davis et al., 2018) was used by employing the frequency-based identification method (i.e., odd high abundance of specific taxa on low DNA-concentration samples) with a threshold of 0.1. This resulted in a total detection of 554 contaminant sequences, corresponding to 46 OTUs, which were removed from the dataset before subsequent analyses.

### LDChip Immunological Analysis for Biomarker Profiling

Fluorescent sandwich microarray immunoassays (FSMIs) were performed on site with the Life Detector Chip (LDChip) within 24 h after sampling, by analyzing crude extracts from 0.5 g of sample as described before (Rivas et al., 2008; Parro et al., 2011; Sanchez-Garcia et al., 2018). This analysis allows the identification of biological polymers (including lipo/exo-polysaccharides), conserved proteins, and peptides involved in key metabolisms (e.g., nitrogen cycling, sulfate reduction, and other energy and iron metabolisms) and principal taxonomic lineages of bacteria and archaea (Rivas et al., 2008; Parro et al., 2011). The list of the 200 antibodies and their corresponding immunogens used in this study are described in Sanchez-Garcia et al., 2018, and the methodology for the FSMI with the LDChip in Blanco et al., 2017; see also Supporting Information Figure S4.

### Statistical Analyses

All statistical tests and analyses were calculated with R language for statistical computing (R Core Team, 2019), including the use of ‘vegan’ v.2.4–3 (Oksanen, 2017) and ‘MASS’ (Ripley et al., 2013) packages.

Shapiro-Wilk tests were employed to assess normality of the parameters included in the study. Statistical differences among analyzed parameters were then tested by means of Mann-Whitney-Wilcoxon (two levels of variance, i.e., nunatak and slope of sampling site) or Kruskal-Wallis (three levels of variance, i.e., altitude of sampling site or substrate sampled) tests, followed by post-hoc Mann-Whitney-Wilcoxon pairwise comparisons when needed (statistical differences found among the three levels of variance). Only those environmental parameters that showed significant differences for at least one of the categories of comparison (i.e., nunatak, slope, altitude, or substrate of sampling point) were employed for subsequent analyses.

Non-metric multidimensional scaling (NMDS) based on Bray-Curtis distances was also calculated and visualized only for physicochemical parameters (ICP-MS and IC) of the lithic substrates, thus allowing to explore similarities between sampling sites composition. Standardized and log-normalized data were employed to carry out this analysis, in order to diminish the influence of zero values on the statistical analysis.

Variability and probability of occurrence of microbial classes on samples was tested by carrying out a logistic regression adjustment (two levels of variance) or a linear discriminant analysis (three levels of variance). For logistic regression, a previous stepwise variable selection was implemented to select the best model for the contrast. Only those classes that showed the highest probability of occurrence were included in subsequent analyses.

Microbial OTU richness (*S*) and Shannon diversity index (*H'*) were calculated. Canonical correspondence analyses (CCA) on microbial classes with environmental parameters and biomarker data were also carried out, followed by a significance test of the CCA model constrains by means of a permutation analysis (999 permutations, function ‘anova.cca’ in ‘vegan’ package). CCA was employed to address which of the constrained environmental parameters are influencing the most the microbial assemblages described by DNA sequencing and LDChip approaches (Ramette, 2007; Paliy and Shankar, 2016).

## Results

### Climatological Analyses

Data retrieved from the Spanish Meteorological Service (AEMET) repository cover a period of almost 31 years since 1988 at the Juan Carlos I automatic weather station and since 2017 at Hurd (Figure 1; Supporting Information Figure S1). Our analyses demonstrated that data for atmospheric variables, such as temperature, relative humidity, and incident radiation strongly correlated between datasets, thus allowing direct extrapolation for longer prior periods at Hurd Glacier. Measurements show colder temperatures and higher humidity at Hurd Glacier station in comparison with the Juan Carlos I station (*r*^2^ > 0.9 during the overlapping years in linear regression analysis, Supporting Information Figure S2). At the Hurd Glacier, monthly mean temperatures range between 0 and 5°C in the summer (from December to March), and from 0°C to −10°C in the winter (from April to October), while humidity is *c*. 80% all year round (Supporting Information Figure S2). Occasionally, northward winds from the Antarctic continent reach Hurd Peninsula causing very cold and dry spells, with temperature and humidity falling below −10°C and 30%. Incident radiation has a seasonal cycle, being comparable among photosynthetically active radiation (PAR), UVA, and UVB (Supporting Information Figure S2). UVA-B radiation (290–400 nm) showed daily maximum peaks of around 100 W/m^2^ at the beginning of January, while UVB erythemal radiation (280–320 nm) showed maximum daily values of around 240 mW/m^2^ in February (Supporting Information Figure S2b). Consequently, the UV radiation index (UVI) also follows this latter cycle, with maximum average daily values between 6 and 8 due to the cloudiness of the island, but reaching values of 14 on clear days, mostly near the summer solstice (Supporting Information Figure S2b). Importantly, these data do not take into account the effect of the surrounding ice and snow, which multiplies the incident radiation from space by reflection.

### Physicochemical Parameters

Geochemical characteristics of the three different substrates were analyzed by X-Ray powder diffraction (XRD, mineralogy, shown in Supporting Information Figure S3), inductively coupled plasma-mass spectrometry (ICP-MS, elemental composition), and Ion chromatography (IC, soluble inorganic anions and low-molecular-weight organic acids) techniques. The highest number of statistical differences for each technique was found when comparing data among different substrates.

ICP-MS analyses showed more than 80 elements present in the samples. Within them, some could have important biological implications, e.g., sodium (Na), iron (Fe), potassium (K), calcium (Ca), phosphorus (P), manganese (Mn), copper (Cu), and bromide (Br; Supporting Information Figure S4). Specifically, Fe, Ca, and Na showed the highest concentrations, but without any trend among samples or substrates.

**Figure 2:**
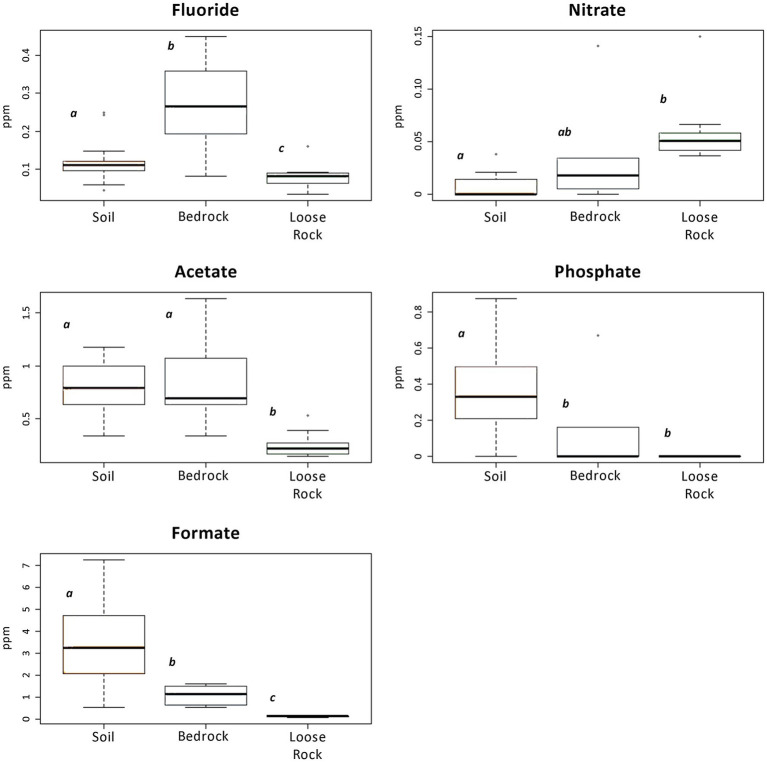
Boxplots presenting ions and organic acids detected by Ion chromatography (IC) analysis that showed any significant difference among the analyzed substrates. Different letters depict significant differences (*p* < 0.05) among the substrates. Note the difference in vertical axes scales.

IC analyses detected the presence of inorganic anions, such as fluoride, chloride, nitrate, phosphate, and sulfate, as well as low-molecular-weight organic acids, such as acetate, formate, tartrate, and oxalate in the samples (Figure 2; Supporting Information Figure S5). Apart from oxalate, fluoride and nitrate, which values were very low on every substrate, most anions were present at a higher concentration in the soil samples in comparison with those of loose sample rocks and bedrock (Supporting Information Figure S5). On average, the most abundant organic acid was formate (up to 7 ppm) in SL-MOO-N1-10 and SL-MOO-N3-10 soil samples (Figure 2; Supporting Information Figure S5). In contrast, chloride concentration (6.05 and 4.06 ppm at R-McG-N3 and R-MOO-S1, respectively) was highest in loose rock samples, while sulfate was highly detected in bedrock samples (16.01 ppm at B-MOO-S3). Despite these trends, only fluoride, formate, nitrate, phosphate, and acetate showed significant differences among substrates (*p* < 0.05; Figure 2). The pH ranged from 5.2 in B-McG-S2 bedrock sample to 8.9 in R-McG-N1 loose rock sample, independently of the substrate or sampling location (data not shown).

**Figure 3:**
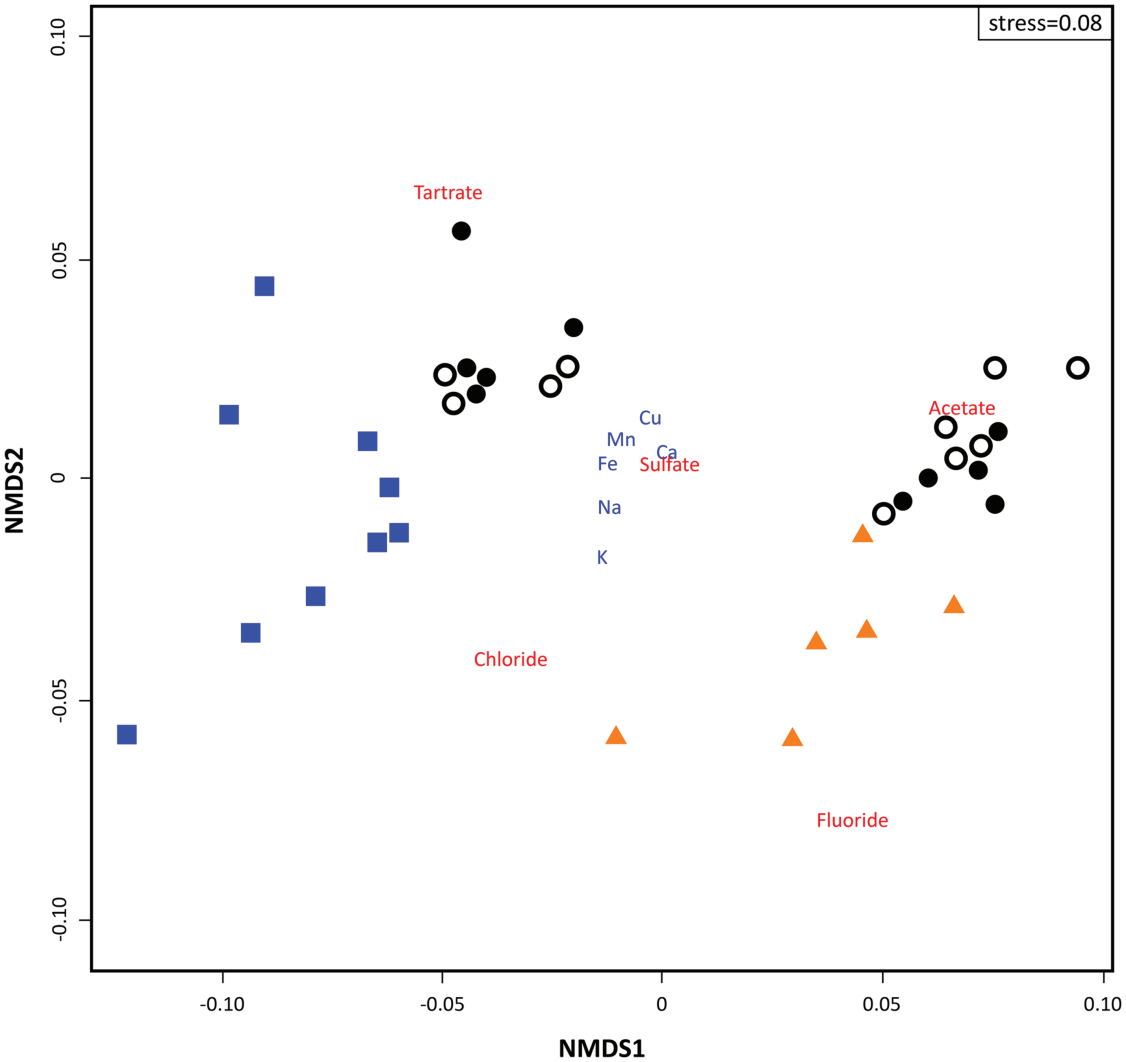
Two dimensional non-metric multidimensional scaling (NMDS) ordination plot of sampling points (based on Bray-Curtis similarities) based on geochemical data analysis (stress = 0.08). Triangles depict ‘Bedrock’ samples; white dots depict ‘Surface soil’ samples; black dots depict ‘10 cm-depth’ samples; and squares depict ‘Loose rock’ samples. ICP-MS data appear in blue font and IC in red.

The NMDS calculated on Bray-Curtis distances for physicochemical parameters (ICP-MS and IC) illustrated two different groups of samples: (1) all loose rocks and nine soil samples at the left of the ordination and (2) all bedrock samples and the rest (*n* = 11) of soil samples at the right (Figure 3). The loose rock samples did not group based on rock type, i.e., volcanic or sedimentary rocks were not more closely located in the ordination attending to its geological origin. Bedrock samples were situated close to fluoride and chloride, while soil samples clustered nearby to organic acids, with two clearly segregated subclusters related to acetate (right of the ordination) and tartrate (top left of the ordination; Figure 3). No other trend related to location of sampling site was observed for any of the samples.

### Microbial Community Composition and Diversity

**Figure 4:**
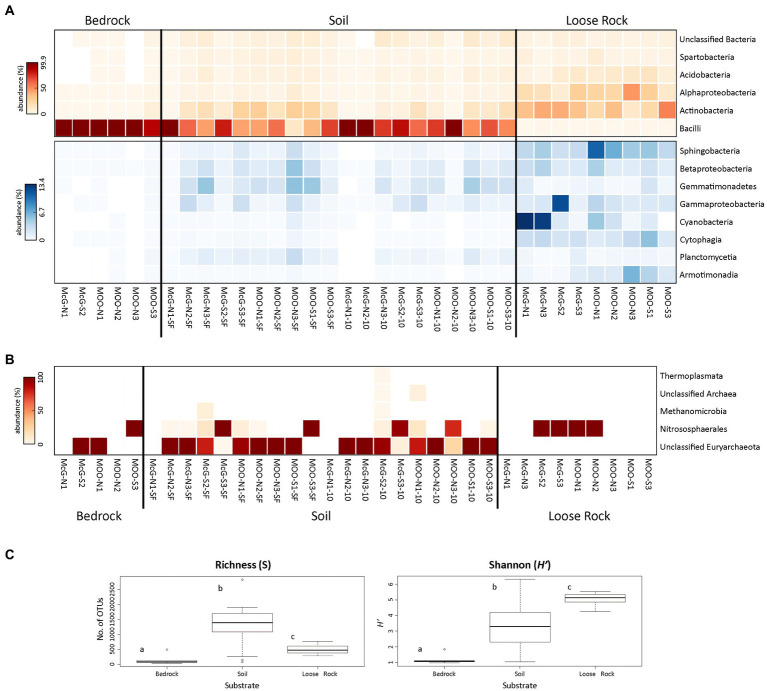
Distribution and diversity of prokaryotic communities among sampling points and substrates. Heat maps showing the relative abundance (number of sequences at the class level) of **(A)** Bacteria and **(B)** Archaea (bedrock sample B-MOO-N3 did not yielded any sequence that was classified as Archaea and, subsequently, was not included in the figure). Only prokaryotic classes accounting for >0.5% of total number of sequences are plotted. Bacterial heat maps are split into two to better show the relative abundance of the less represented classes (blue scale). In both the red and blue scales, white color indicates absence of sequences (please notice the different % of abundance in each heat map). **(C)** Boxplot presenting the Richness (S) and Shannon diversity (*H'*) indices for prokaryotic communities at each of the analyzed substrates. Different letters depict significant differences (*p* < 0.05) among the substrates.

Analyses of 16S rRNA Illumina sequencing of the prokaryotic community showed that the highest number of significant differences (*p* < 0.05) was found among the three analyzed substrates (Figure 4; Supporting Information Figure S6). Bacterial communities showed Bacilli class (Firmicutes phylum) being the most represented taxon in both bedrock (abundance of 99% of total sequences in almost all bedrock sites) and soil communities (abundances from 13.2% in SL-MOO-N3-SF to 99.6% in SL-McG-N1-10). Actinobacteria and Alphaproteobacteria dominated loose rock samples (highest abundances of 53.3% in R-MOO-S3 and 46.9% in R-MOO-N3, respectively, Figure 3). On average, loose rocks contained the most diverse prokaryotic community according to the Shannon diversity index (*H'* = 5.05 ± 0.42), while soil samples on average contained the highest richness values on average (*S* = 1280.3 ± 697.06). Differences across the three substrates for both indices were found to be statistically significant (*p* < 0.05, Figure 4C). Moreover, most bacterial genera (30.8%) were shared among the three substrates (Supporting Information Figure S7), with soil samples showing the highest percentage of unique ones (i.e., present in only one substrate, 22.1%) and bedrock samples the lowest (0.7%).

Bedrock bacterial communities were amply dominated by the Paenibacillaceae family (Bacillales order, Bacilli class), with *Brevibacillus* and *Paenibacillus* being the most represented genera by far (68 and 28% of total sequences on average, respectively). Apart from Bacillales taxa, Solirubrobacterales, Actinomycetales, and Acidimicrobiales orders (Actinobacteria) were also detected, mostly in B-MOO-S3 sample, but in a much lesser abundance (highest abundances of 2.9, 1.9, and 1.5%, respectively, in that sample, Figure 4A).

Soil bacterial communities were similar in composition to those in bedrock samples. Therefore, *Brevibacillus* and *Paenibacillus* (Bacillales order, Bacilli class) were the most abundant genera within this substrate, but with lower average abundances of 49.2 and 15.8%, respectively. Together with the Bacilli class, Actinobacteria was also represented in soil samples, accounting for an average of 12.5% of total sequences (Figure 4A). Solirubrobacterales, Acidimicrobiales, and Actinomycetales, with an average of 4.1, 3.2 and 3.1% of total number of sequences, were the most represented orders within the Actinobacteria class. Acidobacteria, Spartobacteria, Gemmatimonadetes, and Gamma- and Betaproteobacteria classes were also widely found across soil samples, but without any recognizable trend (Figure 4A).

Bacterial communities in loose rock samples showed a different structure compared to those in bedrock and soils. While Bacilli taxa were hardly present, Actinobacteria (average 32.7% of total sequences) and Alphaproteobacteria (22.6%) classes appeared to be much more abundant in these samples (Figure 4A). Members within Solirubrobacteraceae, Actinomycetaceae and Anacardiaceae families (27.2% of total sequences together on average, belonging to Solirubrobacterales and Actinomycetales order, Actinobacteria class), and Acetobacteraceae and Sphingomonadaceae families (19.3% of total sequences on average, belonging to Rhodospirillales and Sphingomonadaceae orders, Alphaproteobacteria class) were the most abundant taxa at these loose rock samples, but without showing any trend related to sampling point or slope. Cytophagia and Beta- (especially Burkholderiales order) and Gammaproteobacteria classes were also widespread in loose rocks, but also without any clear trend. In contrast to bedrock and soil samples, Sphingobacteria and Cyanobacteria classes were found to be abundant in loose rock samples, reaching up to 10.9 and 13.4% of total sequences in R-MOO-N1 and R-McG-N1, respectively. Additionally, while sphingobacterial sequences were found to be more abundant at the Moores nunatak, cyanobacterial ones showed a higher abundance at northern slopes, especially at the McGregor nunatak (Figure 4A).

Archaea were also detected by DNA sequencing in the three analyzed substrates, although these analyses yielded a much lower number of reads in comparison with bacteria. Archaeal sequences mainly belonged to the Euryarchaeota and Thaumarchaeota phyla, with 46.5 and 26.8%, respectively, on average of total Archaea sequences. Unclassified sequences within Euryarchaeota and members of Nitrososphaerales class (Thaumarchaeota) were the most abundant groups, with the first being highly detected in soil and bedrock samples and the latter being the unique group detected in loose rock substrate (Figure 4B). However, lower taxonomic adscriptions remained unknown, as comparison against several databases (Supporting Information Figure S3) yielded a vast majority of unclassified reads. Of special interest was the soil SL-McG-S2-10 sample, where all the detected archaeal groups were present, including Thermoplasmata (unique to this sample) and Methanomicrobia classes.

**Figure 5:**
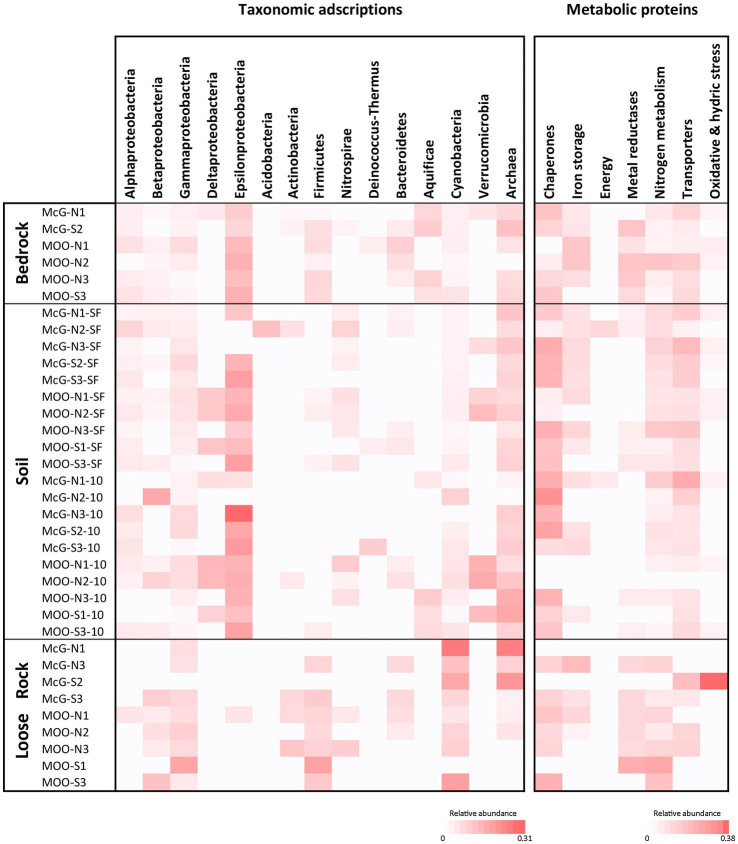
LDChip results distribution heat maps of prokaryotic classes and microbial metabolism biomarkers among sampling points and substrates by means of LDChip analyses. The antibodies recognized by LDChip for microbial taxonomy – further information in Sanchez-Garcia et al. (2018) – were reorganized on the basis of the phylogeny of the targets. Average signal intensity corresponding to positive antibody reactions within each category was used as a proxy for relative abundance (note the different scale in relative abundance under each heat map). For both heat maps white color indicates non-detected signal, while intensity of positive signals is indicated from light pink (lower signal) to red (higher signal).

Qualitatively, immunological analyses with LDChip showed a slightly different community composition than the one obtained by DNA sequencing (Figure 5). However, as well as in DNA sequence analyses, major differences could only be observed among substrates. Epsilon- and Alphaproteobacteria – mainly *Arcobacter* and *Acidiphilium* genera, respectively – were much more detected in soil and bedrock samples compared to loose rock ones (Figure 5). On the contrary, the Firmicutes phylum (mostly members of Bacilli and Clostridia) was amply detected in loose rocks and bedrock samples, while they were absent from most of soil samples. Other bacterial groups, such as Gamma- (*Acidithiobacillus* sp.) and Betaproteobacteria (Rhodocyclales), were also widely detected by LDChip, as well as Cyanobacteria (all terrestrial N-fixers, especially abundant at loose rock samples) and Archaea (mostly Halobacteria and Methanomicrobia).

### Organic Matter and Metabolic Biomarker Profiling

Differences in TOC and TN contents were observed for the three substrates (Supporting Information Figure S8). Overall, soil samples showed relatively higher content of TOC (0.17 ± 0.13%) with a maximum of 0.54% at SL-McG-S3-10 (Supporting Information Figure S8a). In comparison, average TOC contents in bedrock and loose rocks were one order of magnitude lower (0.08 ± 0.04% and 0.05 ± 0.03%, respectively). Similarly, TN was only detectable in the soil samples (0.012 ± 0.016%; maximum of 0.048% at SL-McG-S3-10, Supporting Information Figure S8b).

Stable carbon isotopic composition varied among all samples from −23.0 to −31.6‰ δ^13^C (Supporting Information Figure S8a). The loose rocks were overall most depleted (−28.3±2.2‰), particularly in R-McG-N1 and R-MOO-S1 (−31.6 and −31.3‰, respectively). In contrast, samples of bedrock B-MOO-S3 (−23.3‰) and soils SL-McG-N3-SF and SL-McG-N3-10 (−23 and −23.1‰, respectively) showed the most enriched δ^13^C ratios. The δ^15^N ratio could only be measured in the few soil samples where TN was detected and displayed values from −7.7 ‰ (SL-MOO-S3-SF) to −4.4‰ (SL-MOO-N1-10; Supporting Information Figure S8b).

LDChip immunoassay revealed some metabolic processes common to all samples, and others characteristic only of specific substrates (Figure 5). Signals from thermal shock proteins (chaperones, such as HscA, a member of the Hsp70 protein class) were generally detected in all samples, as well as proteins related to nitrogen metabolism (NifH and NifD, involved in nitrogen fixation and GlnB as regulator of nitrogen assimilation; Figure 5). Transport proteins, mainly for K^+^ cation transport, were also widely detected at soil and bedrock samples, but not in all loose rocks. Similarly, starvation response proteins (PhaC1 protein, involved in the conversion of carbon to PHA) were more distributed within soil and bedrock samples, whereas less abundant in the loose rocks (included among stress proteins in Figure 5). On the other hand, Fe^3+^ and Cu^2+^ metal reductases were hardly found in soils, but broadly detected in loose rock and bedrock samples.

### Geomicrobiological Parameters Ordination

**Figure 6:**
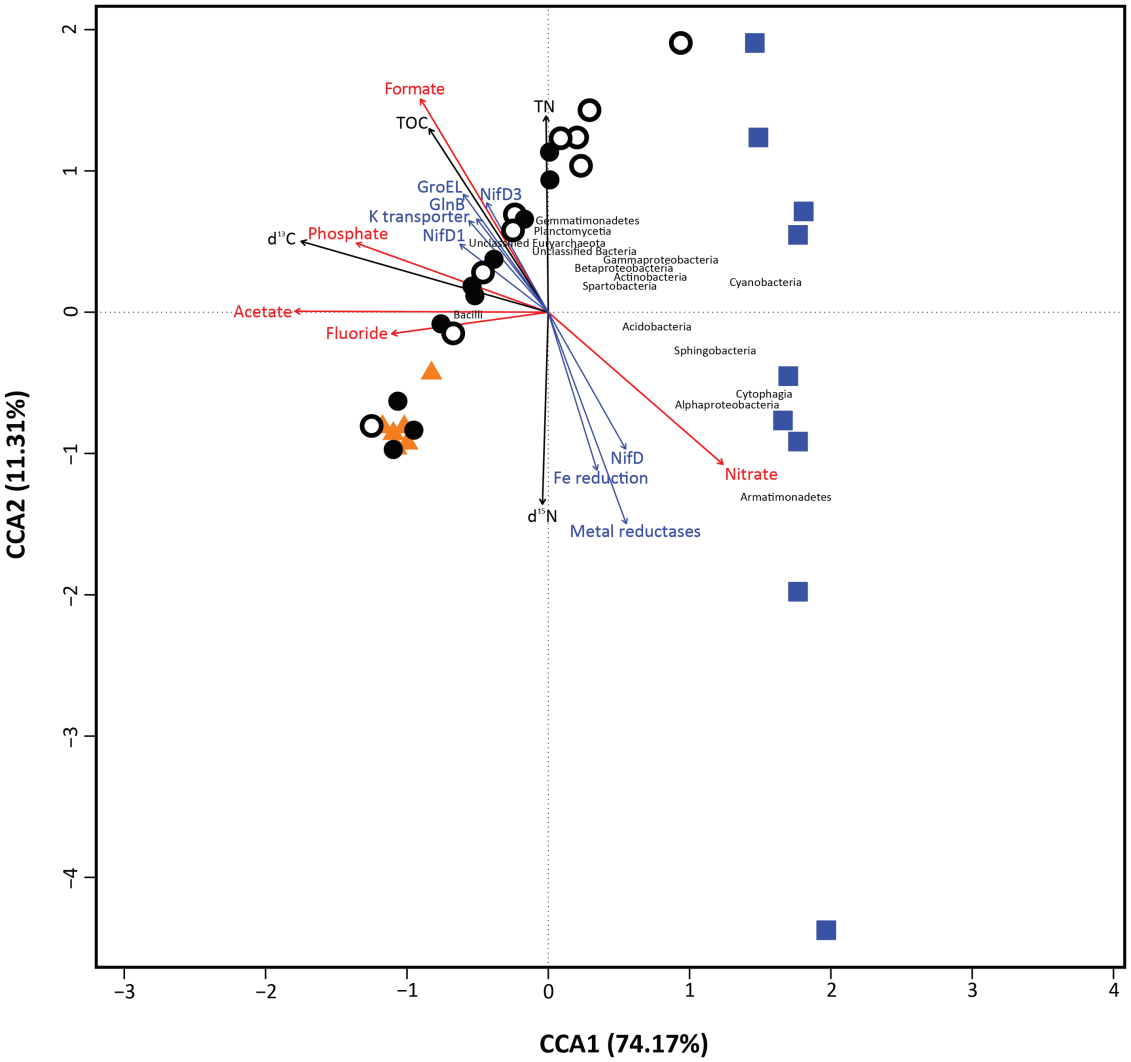
Canonical correspondence analysis (CCA) plot, showing the correspondence of main environmental factors (red arrows represent ion chromatography data) and biomarkers detection (Black arrows depict ‘bulk isotope’ data; blue arrows depict ‘LDChip’ data for microbial metabolism) with the microbial community composition of the samples (Orange triangles depict ‘Bedrock’ samples; white dots depict ‘Surface soil’ samples; black dots depict ‘10 cm-depth’ samples; and blue squares depict ‘Loose rock’ samples. microbial classes appear in black font). The analysis showed 85.48% (CCA1=74.17%, CCA2=11.31%) of total variance of the data and was significant overall at *p* < 0.05 (999 permutations test for CCA).

Canonical Correspondence Analysis (CCA) was employed to show the influence of the measured environmental factors that explain a maximum of the variability on microbial community data (Figure 6). Overall, the analysis explained a total 85.48% of variance in the data (CCA1 = 74.17%, CCA2 = 11.31%; *p* < 0.05), showing two main groups of samples split along CCA1 axis. Soil and bedrock samples were settled on the left of the ordination, while loose rock ones were located to the right of it. Along CCA2 axis, however, soil and bedrock samples grouped closely, together with Bacilli, Gemmatimonadetes, Planctomycetia, and unclassified OTUs from Euryarchaeota and Bacteria, while loose rock samples were located widespread. These loose rocks samples did not show specific grouping based on rock type (e.g., volcanic or sedimentary rocks were not clustered together), but specific samples presented unambiguous relations with certain microbial classes. This is the case of two sites (R-McG-N1 and R-McG-N3) with Cyanobacteria or Cytophagia and Alphaproteobacteria (R-MOO-N2, R-McG-S3 and R-MOO-N1). The rest of microbial classes (such as Bacilli, Actinobacteria, Spartobacteria, and Beta- and Gammaproteobacteria, which were more widely found among the samples) was settled in between the groups close to the center of the ordination.

Different trends, coincident with the distribution of the two groups of samples mentioned above, were found for anions and biomarkers (TOC, TN, stable isotope ratios, and proteins from LDChip; Figure 6). First, organic acids (formate and acetate) and inorganic anions (phosphate and fluoride) were closely located among them and to the soil and bedrock group. Nitrate was the only anion rather related to the loose rocks. Similarly, proteins found by the LDChip were mainly related to the soil group (most of nitrogen cycling proteins together with GroEL chaperonin and a K^+^ transporter), except for NifD and a Fe reduction protein that were rather related to loose rock samples. The stable isotopic ratio δ^13^C and the TN content closely related to each other and to soil samples.

## Discussion

### Geochemical Patterns Among the Three Different Substrates Analyzed at the Antarctic Nunataks

Geochemical analyses of Hurd Peninsula habitats are very scarce in the literature, with soil being the only substrate that has been extensively studied before (Haus et al., 2016; Garrido-Benavent et al., 2020). We therefore extend these studies to nunataks present at this area, also including the study of the uncharted lithospheric substrates in the area, i.e., bedrock as well as loose rocks. Despite their different settings and origin, all lithic substrates showed a very consistent mineralogy mainly based on quartz and plagioclases, as identified by XRD analyses (Supporting Information Figure S3). Our results also revealed pH values close to neutrality, in accordance to those previously described for most developed soils at the Hurd Peninsula (Garrido-Benavent et al., 2020). In contrast, average values of TOC (from 0.17 ± 0.13% in soils to 0.05 ± 0.03% in rocks) and TN (0.012 ± 0.016%, only detected in soils) found for the three analyzed substrates (Supporting Information Figure S8) were only similar to those described in much less developed soils from the terminus of Hurd Glacier (free of ice for only 1 year), very close to our area of study. TOC and TN contents from other Antarctic soils ranged from an order of magnitude lower (0.0058–0.081 and 0–0.020%, respectively), such as those in McMurdo Dry Valleys (Matsumoto et al., 2010), to much higher values (maximum of 5.6% for TOC and 0.46% for TN), such as the closer areas of Cerro Caliente, in Deception Island (Lezcano et al., 2019) or King George Island (Pan et al., 2013). Although similar environmental parameters are described for the islands within the South Shetlands archipelago, TOC, and TN values found at Hurd Peninsula nunataks are more comparable to those found for Dry Valleys soils – subjected to much more severe conditions, especially in terms of low water content and temperature. For all the aforementioned findings, we propose that the specific harsh environmental conditions at nunataks slopes may be greatly hindering primary production by microbial communities and, therefore, the subsequent input of nutrients (such as the measured C and N) to the different substrates. However, the detected values of TOC and TN are compatible with a current, although limited, microbial autotrophic (fixation) origin, similarly to the situation in the Dry Valleys. Low-molecular-weight organic acids (formate, acetate, and tartrate) detected in nunatak soil samples may be then used as reducing power for other microbial metabolisms.

The statistical ordination based only on ICP-MS and IC data (Figure 3; Supporting Information Figures S4, S5) clearly clustered samples attending to the lithic substrate they came from, even showing a high compositional variability within each set. Although there is a remarkable intragroup heterogeneity in bedrocks and loose rocks, variability is even more noticeable for soil samples, which split into two clearly different groups. This is in accordance to the previous findings, since it has been broadly proposed that soil structure often overlaps local-scale gradients on top of regional patterns (Ettema and Wardle, 2002). Loose rocks were described as coming from different rock types (mainly volcanic and sedimentary) and showed different characteristics in terms of porosity and crystalline textures even within the same nunatak slope and rock type, but similar mineralogical composition (Supporting Information Figure S3). Thus, the existence of physically and chemically divergent microhabitats within them seems to rather be the most plausible factor for the divergence observed in the ordination. These microhabitats may result from the physical weathering on these rock substrates due to freezing temperatures, strong winds, and UV radiation (Lee et al., 2004), a process that has a remarkable importance from the astrobiological perspective, since it has been suggested as responsible for the formation of the Martian regolith (Chevrier et al., 2006; Kolb et al., 2006; Viles et al., 2010; Ehlmann and Edwards, 2014); moreover, it could lead to a differential microbial colonization (Archer et al., 2016). On its behalf, a further geological characterization of bedrock at these nunataks would be very valuable to find any possible counterparts on Martian geology.

### Substrate Characteristics Also Explain Principal Differences in Microbial Community Structure and Biomarkers Distribution

We inferred the composition of the microbial communities in the three analyzed lithic substrates by DNA and LDChip analyses. Both approaches detected Archaea, a domain that has not been extensively identified, neither isolated nor cultured, in other polar environments and other Antarctic soil or rocky ice-free areas (Niederberger et al., 2012; Cowan et al., 2014; Crits-Christoph et al., 2016; Bendia et al., 2018; Lambrechts et al., 2019; Ortiz et al., 2020). We retrieved a relative large number of DNA sequences mainly from unclassified Euryarchaeota and Nitrososphaerales (Thaumarchaeota; Figure 4), similarly to other studies carried out in glacier forefields on the nearby King George Island (Pessi et al., 2015) or even in harsher Antarctic environments (Richter et al., 2014; Ortiz et al., 2020; Zhang et al., 2020). On its behalf, LDChip showed positive immunodetections with antibodies to members of Halobacteria and, mainly, Methanomicrobia (Figure 5), two phylogenetically close-related groups of Euryarchaeota (Sorokin et al., 2017; Aouad et al., 2018). As the coexistence of these archaeal groups may be intriguing due to their opposed environmental requirements (i.e., aerobic *vs*. anaerobic conditions), the observed heterogeneity within the lithic substrates may create both aerobic and anaerobic micropores in just a few millimeters. Although comparison between techniques can only be made qualitatively, the complementarity of DNA sequencing and LDChip approaches is proven again by this improved taxonomic adscription of Archaeal sequences. This complementarity is based on the potential of LDChip to recognize other cellular structures apart from DNA (e.g., proteins, lipids, and membrane polysaccharides) which also hold taxonomic information. In this study, the deeper characterization allowed to complete the partial taxonomic adscription of the archaeal community coming from the lower quality of the sequences and the impossibility to find appropriate counterparts in the databases (similarly to Pessi et al., 2015; Zhang et al., 2020). Together, they also point to a high suitability of both open (i.e., sequencing) and close (i.e., immunoassays) techniques to describe microbial communities from extreme environments, despite the small divergences that could inherently appear between these approaches (Fernandez-Martinez et al., 2019; Lezcano et al., 2019; Sanchez-Garcia et al., 2020a). However, both techniques lack of an explicit identification of active microorganisms, so future studies would benefit from also including metatranscriptomics or metaproteomics analysis for a better discussion of these results.

In contrast to what we hypothesized, the distribution of microbial communities also appeared to be related to the type of lithic substrate on the ordination analysis instead of the location of sampling site. Despite the influence of stochastic assembly processes on microbial communities cannot be discarded in any environment (Zhou and Ning, 2017; Feeser et al., 2018), the high level of variance within the data explained by the CCA ordination (85.48%, *p* < 0.05, Figure 6) points to a more important influence of the environmental factors on structuring the nunataks microbial communities, as it has been also suggested for most of Antarctic lithobionts microbial communities (Cary et al., 2010; Bottos et al., 2014; Chong et al., 2015). The grouping of samples observed on Figure 3 also responds to these environmental factors (in that case only elements and ions, as discussed above) in a similar manner, although both plots show slight differences. When the ordination is based on the microbial community composition (Figure 6), loose rock samples showed the highest variability. Soil samples displayed an intermediate variability between this group and bedrock samples – with almost no variation in microbial communities, thus pointing to a negligible effect of any possible different bedrock composition on the communities. This tendency was also supported by the significantly higher Shannon diversity index found for the loose rock samples in comparison with those from the other two substrates. While bedrock substrate is highly sheltered and oligotrophic, soil is a complex and unstable environment that may be more sensitive to temperature fluctuations, freeze-thaw cycles, or transient oxic-anoxic micropores. Loose rocks gather the most appropriate characteristics of both bedrock and soil substrates to be a refuge for less-resistant microorganisms, since these rocks can act both as a shelter against external environmental conditions as well as a reservoir of nutrients (Omelon, 2016; Meslier and DiRuggiero, 2019). The variability in terms of porosity and textures within the different rock types identified also pointed out the existence of a high number of endolithic milder microhabitats in comparison with bedrock and even soil, thus favoring the development of that higher diversity of microbial groups (Archer et al., 2016). Although further efforts are required in future research to measure microclimatic conditions at these same nunataks, the previous works state that, inside rocks, temperature can be between 5 and 15°C above air temperature (Kirby et al., 2012; Amarelle et al., 2019), and extreme UVA and UVB values can be totally mitigated at less than 2 mm deep, even with translucent minerals (Wierzchos et al., 2015). Again, this scenario is in accordance to what has been previously described for lithic communities from other extreme environments, such as different locations within continental Antarctica, including the Antarctic Dry Valleys (De Los Ríos et al., 2007, 2014; Coleine et al., 2020) and the Atacama Desert (Wierzchos et al., 2018), but in contrast to nearby Antarctic soils subjected to milder conditions, which have been described to harbor a much higher microbial diversity than rocks (Garrido-Benavent et al., 2020).

*Brevibacillus* – dominant genus in both bedrock and soil samples – has been described before to include psychrophilic and rock-inhabiting microbes, which also present a high abundance of genes involved in the response to different environmental stressors (Nicholson, 2002), characteristics that could explain its presence in these two Antarctic substrates while rocks were mainly inhabited by less-resistant taxa. The detection of GroEL chaperonin in these same soil and bedrock samples (Figure 5), together with other chaperones detected by LDChip (Figure 5) which maintain proper protein folding and functionality, also points to the capability of these microbial communities to cope with low temperatures at both substrates, as it has been found before (Alcazar et al., 2010; García-Descalzo et al., 2014). Again, the detection of starvation response proteins, such as Pha1, on these two substrates may also be related to *Brevibacillus* capabilities of coping with these stressors. Moreover, we detected a majority of members within Bacillales order that have also been described to induce sporulation when conditions are adverse (Panda et al., 2014), a feature that would likely take place especially in bedrock samples, due to the aforementioned extreme scarcity of nutrients (Supporting Information Figure S8).

The generally depleted bulk δ^13^C values observed in all samples (from −23.0 to −31.5 ‰, Supporting Information Figure S8) fell within the range of values typically showed by autotrophs incorporating inorganic carbon through the Calvin-Benson-Bassham cycle (i.e., from −19 to −30‰ (Hayes, 2001). We thus concluded that carbon fixation in the three substrate ecosystems occurred dominantly through the Calvin-Benson-Bassham cycle, whereas other autotrophic pathways (e.g., reductive acetyl-CoA resulting into δ^13^C values from −28 to −44‰; (Preuß et al., 1989) were most likely minority. The range of δ^15^N observed in the soils (from −4 to −7 ‰) suggested fractionations related to N_2_ fixation *via* nitrogenase (Robinson, 2001), consistent with the widespread presence of nitrogen fixing proteins detected by the LDChip. Therefore, we are able to suggest photosynthetic and diazotrophic microorganisms may play a pivotal role in the ecosystem as primary producers, thus permitting an active microbial cycling at these nunataks. Bulk δ^13^C or δ^15^N ratios, however, do not allow distinguishing between ancient and active metabolisms. Thus, based on the relatively fast turnover of carbon for an extremely cold environment reported in the Dry Valleys (Hopkins et al., 2009), we interpret the measured δ^13^C values as most likely related to active and/or recent carbon fixation processes, although contribution from ancient metabolisms cannot be completely discarded in anyway.

On the other hand, heterotrophic metabolisms, making the most of the low TOC content present in soils (probably coming mainly from the abovementioned primary production, Supporting Information Figure S8), would have led to the formation of the low-molecular-weight organic acids detected, such as formate and, subsequently, acetate (Supporting Information Figure S5). This process probably might be a consequence of mixed acid fermentation metabolism in the transient anoxic soil microniches, which could take place after, for instance, snow melting. This fermentation process can be carried out by different members of the genus *Brevibacillus* or other groups within Firmicutes phylum (Penning and Conrad, 2006; Yao et al., 2020). In turn, formate and acetate could be substrates for methanogenesis in transient anaerobic niches – potentially carried out by the detected archaeal groups – and sulfur oxidation when transient aerobic conditions are present, as it has been also proposed previously (Ortiz et al., 2020). These findings would need to be discussed in future studies focusing on currently active metabolisms.

The microbial community in the loose rock substrate was composed by taxa mainly found before in soils from other close but milder environments (Garrido-Benavent et al., 2020) and other Antarctic endolithic settings (de la Torre et al., 2003; Archer et al., 2016). While resistant Bacilli were almost absent, we detected significantly higher abundances of heterotrophic Actinobacteria and Alphaproteobacteria. Although with low biomass, loose rocks showed evidence of carbon fixation metabolisms (see δ^13^C values) and primary producers, such as Cyanobacteria, were also greatly detected in these samples, similarly to what has been stated before (Cowan et al., 2014). This scenario is especially remarkable at northern slopes of the nunataks, where a higher sunlight insolation is received and a warmer microclimatic conditions may be expected relative to the southern slopes; this may be indicating a preference for cyanobacterial communities to colonize sheltered, illuminated, milder microhabitats. This preference has been previously described at extreme environments: microbial communities in the Antarctic Dry Valleys (Archer et al., 2016; Van Goethem et al., 2016) and at a completely different area, such as the Atacama Desert (reviewed by Wierzchos et al., 2018), showed this same distributional pattern. Further, Cyanobacteria could also be contributing to nutrient cycling by nitrogen input at this loose rocks substrate by means of nitrogen fixation to ammonia, pointed out by the detection of some related proteins (Figure 5), in a similar way as it has been previously found for other Antarctic environments (Cowan et al., 2014). Consequently, nitrification could be then carried out by the abundantly detected *Burkholderiales* and *Sphingobacteriales* genera, all of them thus responsible of the higher values of nitrate measured relative to the other two substrates (Figure 2; Supporting Information Figure S5). As discussed above, further investigation on current microbial activities, by means of metatranscriptomics and metaproteomics approaches, would be very helpful to determine how active these communities really are.

### The Environmental Setting of Antarctic Nunataks as Analog of Cold Early Mars

It is widely accepted that during the first billion years of Mars geological history, the Noachian period, conditions at the surface of the planet were dominated by very cold temperatures, probably under the freezing point of liquid water. Only specific locations within some circum-equatorial latitudes would have experienced temperatures over freezing, and probably just for a few hours per day during the summer (Fairén, 2010; Fairen et al., 2010), thus allowing the presence of liquid water on the surface of Mars. Although the origin, the climate model or what specific local circumstances allowed that liquid state – even at temperatures below 0°C – is not a consensus (Fairén, 2010; Wordsworth, 2016; Ramirez, 2017), the presence of liquid water points undoubtedly to the moment when life would have had the highest chance to thrive on Mars. Following all of that – and in comparison with other terrestrial Mars analogs that show similarities to current environments on that planet –, is possible to state that climatic conditions described for Hurd Glacier during most of the year could resemble these same milder periods in Martian history (Fairén, 2010). Moreover, since the main climatic conditions on early Mars are assumed to be cold, uncovered terrains, where mentioned liquid water could have appeared, would probably had been surrounded by ice and snow, looking alike ‘exposed rocky islands’ within a glacier, just as terrestrial nunataks are. Thus, both characteristics of the studied nunataks make them a better analog site for studying how life could have developed on Mars in comparison with other Antarctic areas, which show similar conditions to recent Martian environments, when life development might have been even more difficult.

In addition to temperature and humidity, UV radiation on Mars surface – double that of Earth at mid-latitude, including UV-C, a wavelength that does not reach Earth’s surface (Schuerger et al., 2003) – has been also described as a major factor hindering life (Häder and Cabrol, 2018). Following the reflection of solar radiation on surrounding snow and ice – which can be up to 75% or even 100%, respectively (Fioletov et al., 2010) – UV values at nunataks exposed slopes would probably be even much higher than the measured values of 240 mW/m^2^ or 14 UVI at certain moments. Similarly to what could happen on Mars surface, this situation may be forcing microbial communities to adopt different strategies to cope with this hindering for microbial growth. To the best knowledge of the specific conditions faced by the analyzed microbial communities, further measurements should be carried out at those slopes in order to calculate the real UV values to which these communities are exposed, both by incident and reflected radiation. Despite this gap in data, our results point to a preference for endolithic microhabitats – i.e., sheltered from most extreme UV radiation – for a higher diversity of taxa, in contrast to findings in soils from closely located, milder environments (Garrido-Benavent et al., 2020).

Overall, our results regarding the geological features of loose rocks showed that samples were similar to those described before in Miers formation (mainly sandstones, mudstones, and conglomerates; Smellie et al., 2004; Kraus et al., 2008), with the exception of volcanic rocks, such as andesites and tonalites present at the southern slope of McGregor Nunatak and the northern slope of Moores Nunatak. Although a further geological characterization would be needed to classify all substrates, most loose rock types – specially the volcanic ones – may have their counterpart on the surface of Mars (Poulet et al., 2005; Chevrier and Mathé, 2007; Milam et al., 2010). Together with the abovementioned environmental conditions and maximum values of UV radiation, these findings may also reinforce the suitability of Antarctic nunataks as an early Mars analog, particularly for a cold and wet period as the Noachian was.

The opportunity of studying such a different number of biological features – including molecules that can be considered as trustworthy biomarkers – geological and mineralogical substrates also presents on the Mars surface, make Hurd Peninsula an interesting area for astrobiological research not considered before. Thus, our results also help in making a decision in what would be the most accurate technique and scope of a putative Martian search-for-life study, depending on what substrate and biosignature would be the objective of the exploratory mission.

## Conclusions

**Figure 7:**
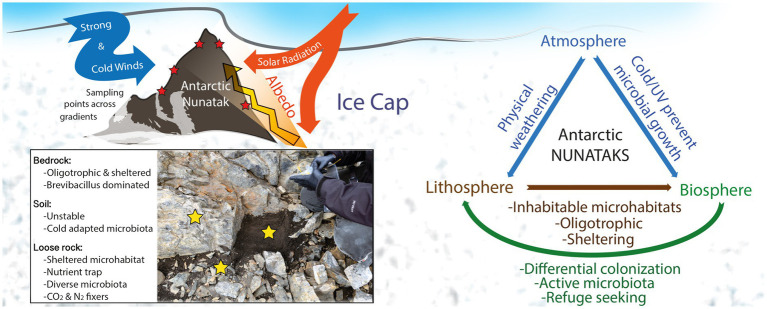
Conceptual map showing environmental situation of nunataks from Hurd Peninsula, Livingston Island, and main findings of the present study.

The specific combination of extreme physicochemical parameters in these unique nunatak environments suppresses most of microbial groups, while the prevailing taxa show different adaptation strategies to cope with these conditions. This results in a microbial community structure more similar to those found in the harsher, far-located Antarctic Dry Valleys than to close-by uncovered terrains. Also, in the confirmation of the suitability of these terrains as potential early Mars analogs. Specifically, high UV radiation, increased by extremely high albedo from surrounding ice cap, together with strong winds and cold temperatures, has both an influence on the lithic substrates and on the abovementioned inhabiting microbial communities (see conceptual map, Figure 7). The physical weathering on the lithic substrates is creating different microhabitats that are then differentially colonized by diverse microbial communities, a feature that results in an overlap of those differences on top of any other local gradient (e.g., altitude, orientation, location), contrarily to what was hypothesized. Bedrock is an extremely oligotrophic, sheltered substrate, soil is highly affected by environmental stochastic phenomena, while loose rocks present a better balance between nutrient accumulation, sheltering, and stability. Thus, bedrock and soil substrates show a community dominated by cold, starvation-adapted, and endospore-forming microbial taxa, such as *Brevibacillus* genus. Meanwhile, loose rocks harbor a highly diverse community, similarly to close soils subjected to milder climatic conditions (Figure 7). Nonetheless, results point to all the substrates may contain metabolically active microbial communities of photosynthetic, chemolithotrophic, and diazotrophic microbes, as depicted by the stable isotopic ratios of δ ^13^C and δ ^15^N and the biosignatures detection by LDChip (Figure 7), although further confirmation will be needed. In addition, the differences among substrates open a field of research devoted to better understanding of what specific biomarkers and, subsequently, techniques should be employed in future space missions to Mars intended to find any sign of life in similar lithic environments.

## Data Availability Statement

The datasets presented in this study can be found in online repositories. The names of the repository/repositories and accession number(s) can be found below: SRA, http://www.ncbi.nlm.nih.gov/sra, PRJNA723417.

## Author Contributions

MF-M: conceptualization, methodology, formal analysis, investigation, writing – original draft, writing – reviewing and editing, and visualization. MG-V: methodology, investigation, resources, and data curation. MM-P: methodology, formal analysis, investigation, and writing – review and editing. VG: investigation, data curation, and visualization. DC and LS-G: investigation, resources, and writing – review and editing. YB: formal analysis, investigation, and writing – review and editing. SG and OP-B: formal analysis, investigation, resources, writing – review and editing, and visualization. IA: writing – review and editing and visualization. LW: conceptualization and writing – review and editing. VP and AF: conceptualization, writing – review and editing, supervision, project administration, and funding acquisition. All authors contributed to the article and approved the submitted version.

### Conflict of Interest

The authors declare that the research was conducted in the absence of any commercial or financial relationships that could be construed as a potential conflict of interest.

## Footnotes

^1^http://www.ncbi.nlm.nih.gov/sra
