# Supplementary material for: Geomicrobiological Heterogeneity of Lithic Habitats in the Extreme Environment of Antarctic Nunataks: A Potential Early Mars Analog
Source: Front Microbiol. 2021 Jul 2;12:670982. doi: 10.3389/fmicb.2021.670982 (PMC8284421; doi:10.3389/fmicb.2021.670982)
Supplement: Supplementary file 1 [file Data_Sheet_1.PDF]

Supporting information to:

Geomicrobiological heterogeneity of lithic habitats in the extreme environment of Antarctic Nunataks: A potential early Mars analog

Miguel Ángel Fernández-Martínez[1,2*], Miriam García-Villadangos[1], Mercedes Moreno-Paz[1], Valentin Gangloff[1], Daniel Carrizo[1], Yolanda Blanco[1], Sergi González[3], Laura Sánchez-García[1], Olga Prieto-Ballesteros[1], Ianina Altshuler[2], Lyle G. Whyte[2], Victor Parro[1] and Alberto G. Fairén[1,4].

[1] Centro de Astrobiología, CSIC-INTA. Carretera de Ajalvir km. 4, 28850 Torrejón de Ardoz, Madrid, Spain.

[2] Department of Natural Resource Sciences, Faculty of Agricultural and Environmental Sciences, McGill University, Ste. Anne-de-Bellevue, Quebec, Canada.

[3] Antarctic Group, Agencia Estatal de Meteorología, Barcelona, Spain.

[4] Department of Astronomy, Cornell University, Ithaca, 14853, NY, USA.

*Corresponding author: mafernandez@cab.inta-csic.es

miguel.fernandezmartinez@mcgill.ca

SUPPORTING INFORMATION S1

Inductively Coupled Plasma-Mass Spectrometry (ICP-MS) methods and conditions

**Component/Parameter**	**Type/value**
Nebulizer	PFA Hight performance HF resistant Type C
Spray Chamber(Peltier-cooled)	PFA Double pass for use with HF
Sampler and skimmer cones	Nickel
Sample Uptake Rate	350μL/min
PF Power	1600 W
Injector	2.0 mm Id quartz
Sweeps	20
Dwell Time	50
Scan mode	Peak hopping
Replicates	3
Mixing Tee	On-line addition of internal standard
KED helium mode	4l/min

The instrument was tuned to maximum sensitivity and the lowest background ,oxides and double charge ion prior to the analysis using a solution containing 1μg/L Be, Ce, Fe In, Li, Mg, Pb and U. Samples were taken up by an ASX-500 CETAC Autosampler and on-line addition of internal standard.

Helium (99.9999%) was used as collision gas (KED mode) to remove possible polyatomic interferences.

A Quality control standard containing 100 ppb of all elements was measured at the end to control the standard recovery and equipment deviation.

SUPPORTING INFORMATION Fig. S1

Overview of sampling sites at Hurd Peninsula

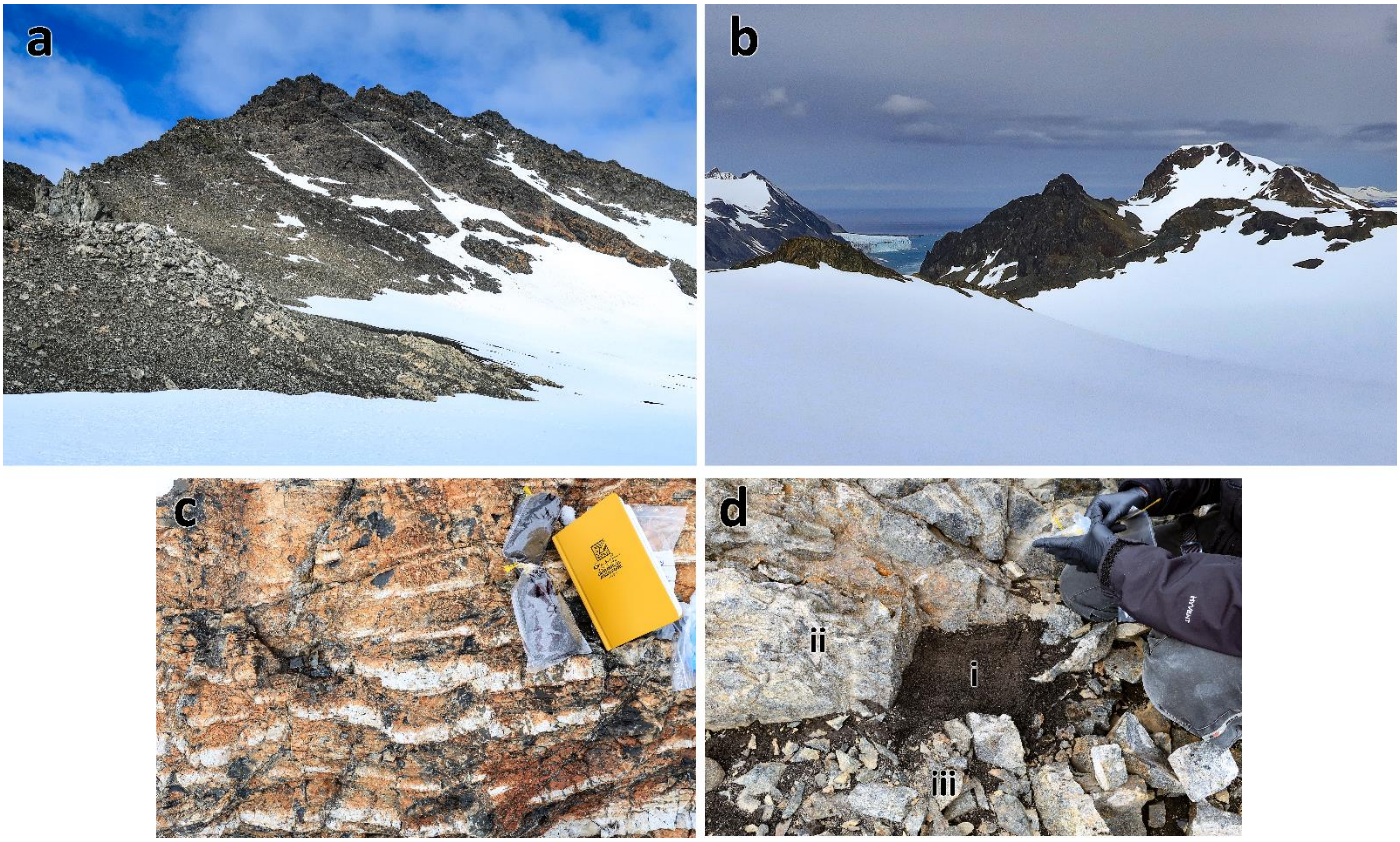

(a) McGregor nunatak from its north slope

(b) Moores nunatak from its south slope

(c) Example of the external aspect of one bedrock sample at Moores nunatak

(d) Example of a sampling point, with: (i) Soil substrate, (ii) Bedrock substrate, and (iii) Loose rock substrate

SUPPORTING INFORMATION S2

Loose Rock DNA extraction

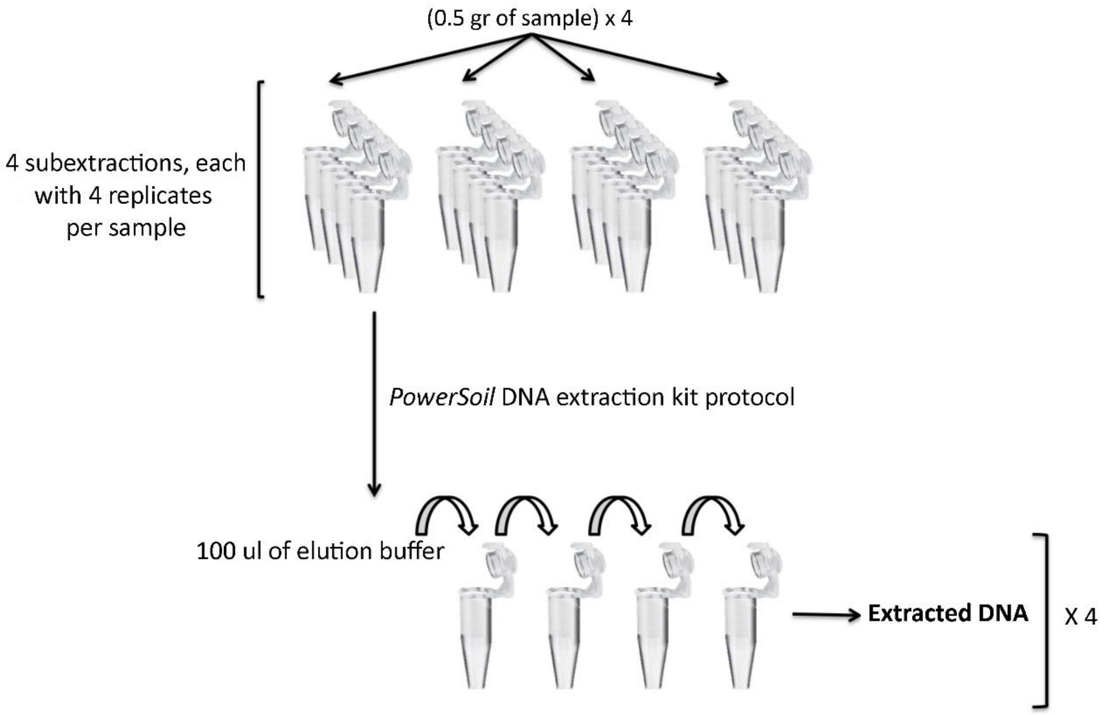

PCR and Illumina protocols from Fundación Parque Científico de Madrid (FPCM)

Purified DNAs were quantified by Picogreen and 200 pg of input DNA were used in the first 'Amplicon PCR' with Q5® Hot Start High-Fidelity DNA Polymerase (New England Biolabs) (26 cycles) in the presence of 100nM primers for bacterial 16S rDNA gene region amplification (341-F / 805-R primer pair) or in the presence of 100nM primers for archaeal 16S rDNA gene region amplification (Arch1F / Arch1R primer pair). After the first PCR, a second 'Index PCR' was performed with Q5® Hot Start High-Fidelity DNA Polymerase (New England Biolabs) (15 cycles) in the presence of 400nM of primers (5'– AATGATACGGCGACCACCGAGATCT-ACACTGACGACATGGTTCTACA-3' and 5'- CAAGCAGAAGACGGCATACGAGAT-[barcode]–TACGGTAGCAGAGACTTGGTCT-3') of the Access Array Barcode Library for Illumina Sequencers (Fluidigm). The obtained amplicons were validated and quantified by an Agilent 2100 Bioanalyzer using DNA7500 chips. Then, an equimolecular pool of these amplicons was purified with AMPureXP Beads (Beckman coulter) to eliminate primers/dimers and titrated by quantitative PCR using the "Kapa-SYBR FAST qPCR kit forLightCycler480" and a reference standard for quantification. The pool of amplicons were denatured prior to be seeded on a flowcell at a density of 10pM, where clusters were formed and sequenced using a "MiSeq Reagent Kit v3", in a 2x300 pair-end sequencing run on a MiSeq sequencer.

SUPPORTING INFORMATION S3

Bioinformatic processing of reads from Illumina MiSEQ sequencing

Reads containing below a minimum number of bp (≤440 bp for bacteria and ≤350 bp for archaea), those containing ambiguous nucleotide identities ("Ns") and/or homopolymers longer than 8 bp, as well as singletons and/or those reads identified as putatively chimeric, were removed from subsequent analyses. Remaining reads were then clustered into 97% sequence similarity Operational Taxonomic Units (OTUs). Sequencing depth for each sample was tested by means of rarefaction curves constructed using iNEXT Online (Chao et al., 2016). Datasets were then independently rarefied to even sequencing depth, corresponding to the lesser number of quality-filtered sequences in the samples, as well as sequences identified as contaminants were removed (resulting in a total of 95,756 sequences for bacteria, 1,637 for archaea) before downstream analyses. Taxonomic affinities were assigned by comparison of OTUs representative sequences against RDP database (RDP v.16 reference files; release 11, Cole et al., 2013). Bacterial OTU's affinities reported as "cyanobacteria/chloroplast" and archaeal OTUs were further assigned a taxonomic identity by comparing them against nr/nt (NBCI), EMBL, Greengenes and SILVA databases for more precise cyanobacterial taxonomic identification. Sequences assigned to mitochondria or chloroplast were removed from further analyses.

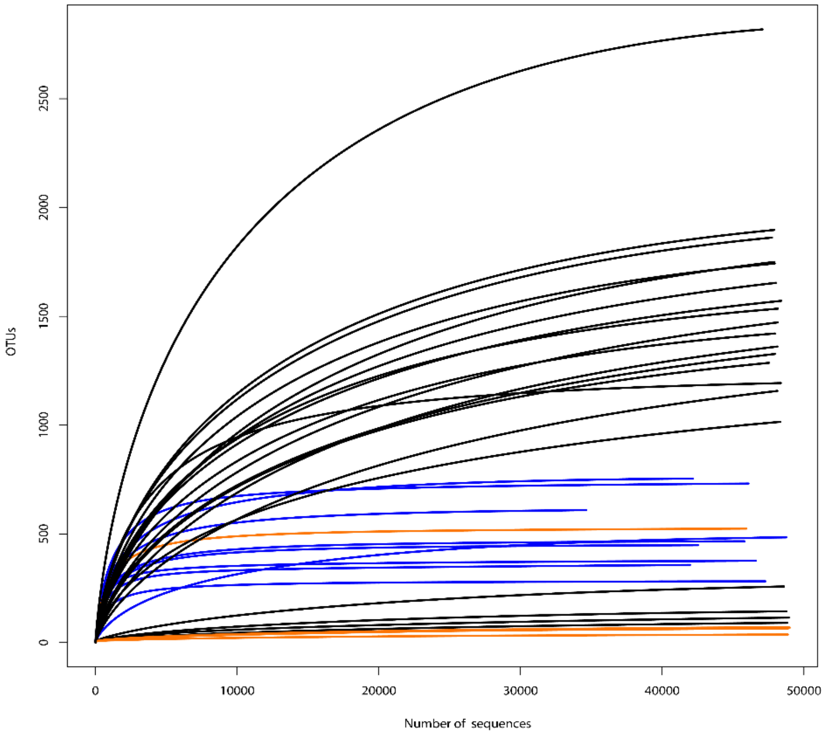

Figure: Rarefaction curves showing that most of soil (in black), loose rock (in blue) and bedrock samples (in orange) reach or approach to plateau, showing sampling effort was sufficient.

Chao A, Ma K, Hsieh T. iNEXT (iNterpolation and EXTrapolation) online: software for interpolation and extrapolation of species diversity. Program and user's guide. chao stat nthu edu tw/wordpress/software_ download. 2016.
Cole JR, Wang Q, Fish JA, Chai B, McGarrell DM, Sun Y, et al. Ribosomal Database Project: data and tools for high throughput rRNA analysis. Nucleic acids research. 2013;42(D1):D633-D42.

SUPPORTING INFORMATION S4

LDChip analysis and data processing

First, IgG fraction of each antibody was printed in a triplicate spot-pattern on the surface of epoxy-activated glass slides as. Another IgG fraction were fluorescently labeled with Alexa 647, titrated, and used in a mixture that consisted of 181 antibodies to reveal the immunoreactions as reported by Rivas et al. (2008). Second, 0.5 g of each soil, bedrock and loose rock samples were resuspended in 2 ml of TBSTRR buffer (0.4 M Tris-HCl pH 8, 0.3 M NaCl, 0.1% Tween 20) as a multianalyte-containing sample for the FSMI. Thirdly, the glass slides were scanned and obtained LDChip200 images were analyzed and quantified by GenePix Pro Software (Molecular Devices, Sunnyvale, CA, USA) as previously reported (Blanco et al., 2012). An additional cutoff value, which was the first interval of fluorescence with an accumulated frequency higher than 80% and an increase less than 10% from the previous one, was applied to minimize the number of false positives. Finally, output fluorescence data were normalized prior to downstream analysis attending to number of probes per taxonomic/metabolic group and to total microarray fluorescence values following He et al. (2007) protocols. Data were classified as a function of the microbial phylogeny of the strain used as immunogen or following main metabolic pathway of the proteins binding the immunogens (see Fernández-Martínez et al., 2019).

Rivas LA, García-Villadangos M, Moreno-Paz M, Cruz-Gil P, Gómez-Elvira J, Parro V. A 200-antibody microarray biochip for environmental monitoring: searching for universal microbial biomarkers through immunoprofiling. Analytical chemistry. 2008;80(21):7970-9.

Blanco Y, Prieto-Ballesteros O, Gómez MJ, Moreno-Paz M, García-Villadangos M, Rodríguez-Manfredi JA, et al. Prokaryotic communities and operating metabolisms in the surface and the permafrost of Deception Island (Antarctica). Environmental microbiology. 2012;14(9):2495-510.

He Z, Gentry TJ, Schadt CW, Wu L, Liebich J, Chong SC, et al. GeoChip: a comprehensive microarray for investigating biogeochemical, ecological and environmental processes. The ISME journal. 2007;1(1):67.

Fernandez-Martinez MA, Dos Santos Severino R, Moreno-Paz M, Gallardo-Carreno I, Blanco Y, Warren-Rhodes K, et al. Prokaryotic Community Structure and Metabolisms in Shallow Subsurface of Atacama Desert Playas and Alluvial Fans After Heavy Rains: Repairing and Preparing for Next Dry Period. Front Microbiol. 2019;10:1641.

SUPPORTING INFORMATION Fig.S2

Climate data for Juan Carlos I Antarctic Station and Hurd Glacier automatic weather stations for the period 1988-2017 (Source: Spanish Meteorological Service, AEMET)

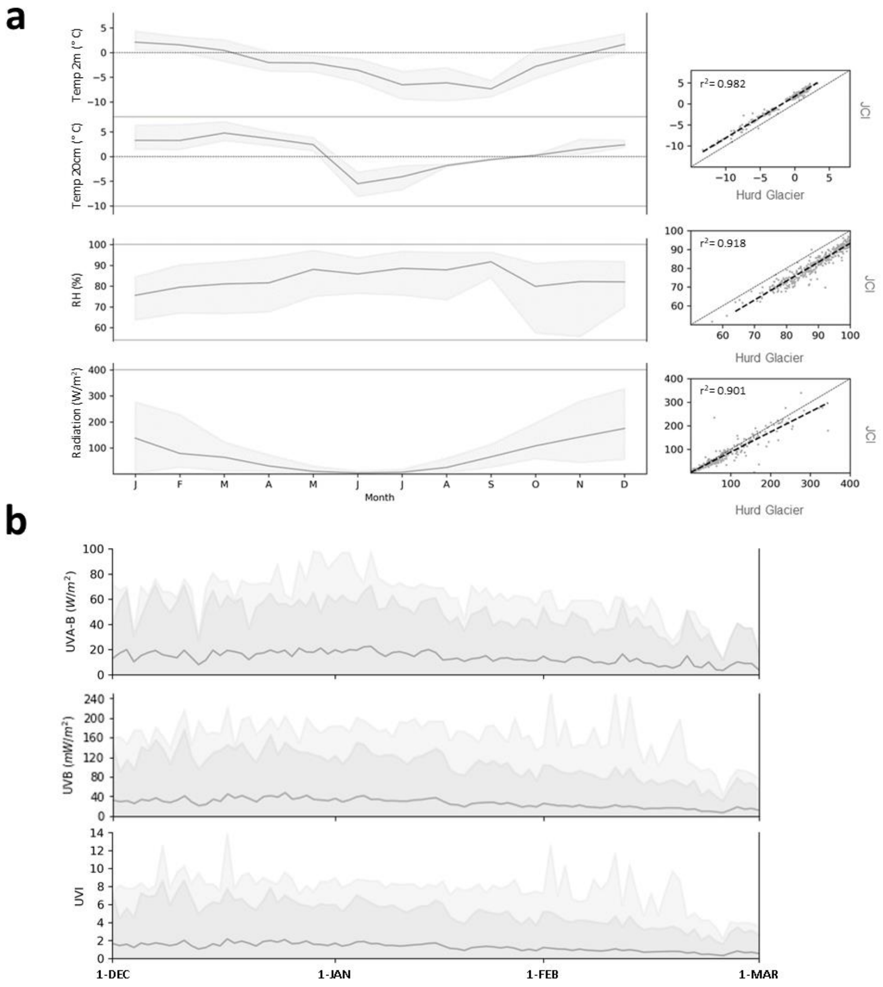

(a) Left graphs show monthly climatological parameters at Juan Carlos I Station. From top to bottom: near-surface temperature at 2 m, near surface temperature at 20 cm, relative humidity at 2 m and surface radiation. Right graphs show correlation between data collected at Juan Carlos I Station and at Hurd Glacier. From top to bottom: near-surface temperature at 2 m, relative humidity at 2 m and surface radiation.

(b) Daily climatology of UVA-B radiation (290-400 nm), UVB erythemal radiation (280-320 nm) and UVI during the summer season (December-January-February).

At both (a) and (b) graphs, solid black lines show the average value of the measured parameter (including day and night measurements), while dark grey areas above and below it show the average highest and average lowest values of it, respectively. For (b) graphs, light grey areas on top show the highest values measured daily for each parameter.

SUPPORTING INFORMATION Fig.S3

X-ray diffraction (XRD) analyses

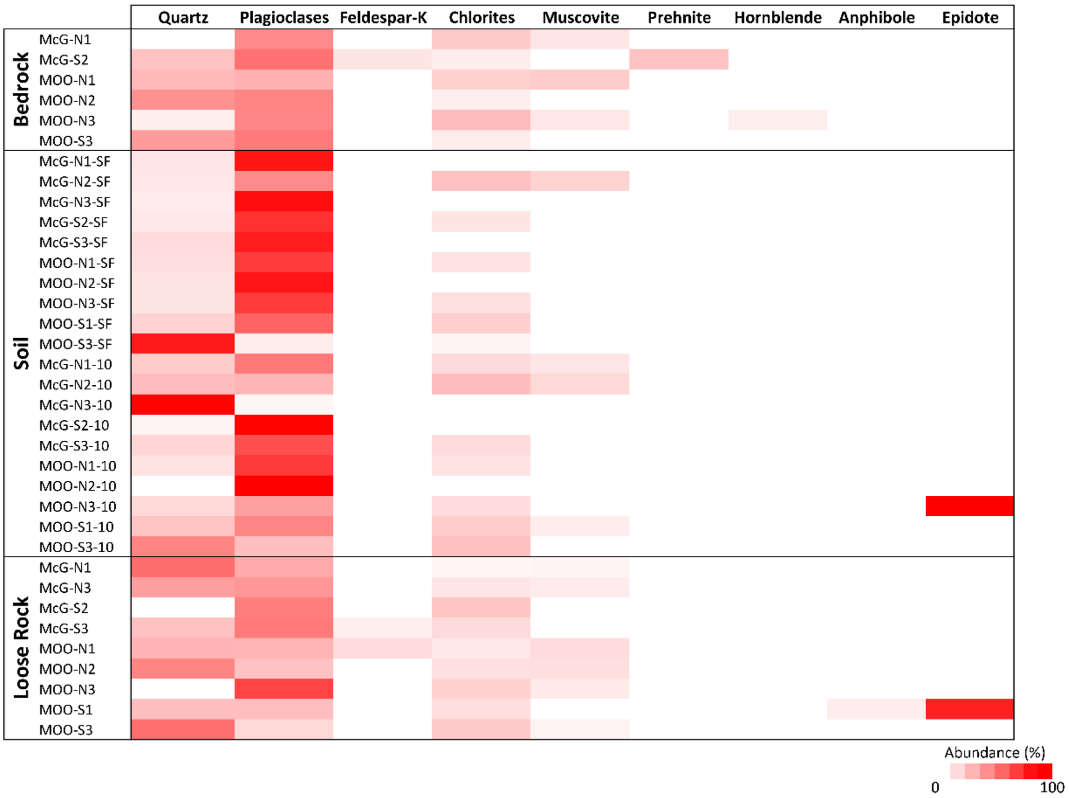

Heatmap showing mineralogical composition (mineral abundance) of soils obtained by powder X-Ray diffraction analyses (XRD). White color indicates non-detected signal, while intensity of positive signals is indicated from light pink (lower signal) to red (higher signal). Muscovite at bedrock sample B-MOO-N3 corresponds to phlogopite and at soil sample S-MOO-S1-10 corresponds to Illite. Loose rock samples are classified as sandstones (R-MCG-N2, R-MCG-N3, R-MOO-N1, R-MOO-N2, R-MOO-S2), andesites (R-MCG-S2, R-MCG-S3, R-MOO-N3), a dike of quartz (R-MCG-N1) and a tonalite (R-MOO-S1).

SUPPORTING INFORMATION Fig.S4

Inductively Coupled Plasma-Mass Spectrometry (ICP-MS) results

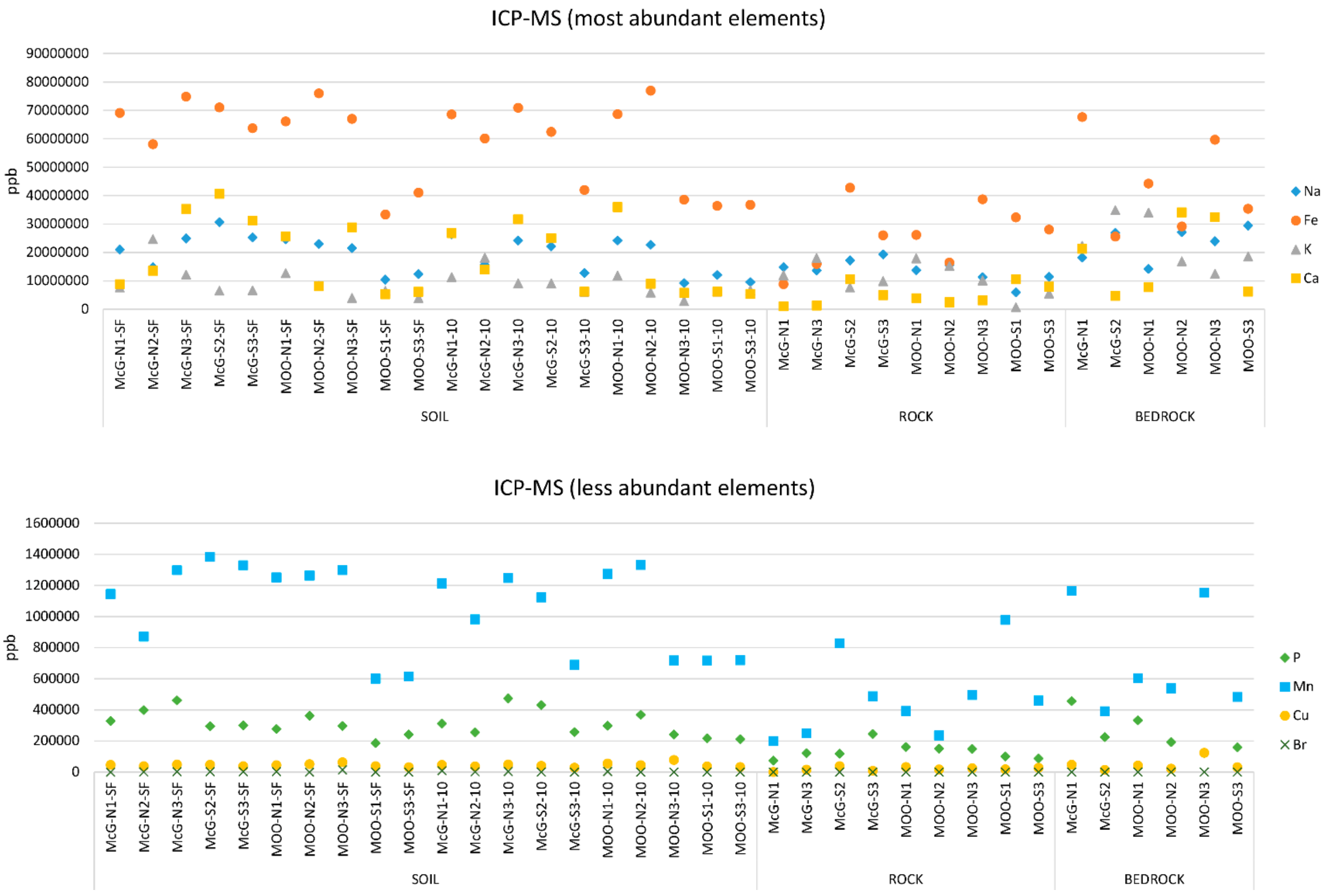

Distribution of elements detected by Inductively Coupled Plasma-Mass Spectrometry (ICP-MS) among the different samples and substrates. Results of the most abundantly detected and microbially related elements were split into two graphs to better show the concentration of the less abundant elements within this group (lower graph, notice the difference in vertical axis).

SUPPORTING INFORMATION S5

Physicochemical composition of substrates obtained by ion chromatography (IC) in ppm

		Inorganic anions										Low molecular-weight organic acids							
	**Sample**	**Fluoride**	sd	**Chloride**	sd	**Nitrate**	sd	**Phosphate**	sd	**Sulfate**	sd	**Acetate**	sd	**Formate**	sd	**Tartrate**	sd	**Oxalate**	sd
**Bedrock**	McG-N1	0.082	0.014	0.592	0.225	0.034	0.011	0	0	0.726	0.015	0.339	0.015	1.496	0.015	0	0	0.015	0.005
	McG-S2	0.260	0.021	0.574	0.025	0.008	0.005	0.669	0.002	0.980	0.001	0.631	0.114	0.505	0.024	0.056	0	0	0
	MOO-N1	0.449	0.032	0.324	0.015	0.005	0	0.158	0.007	1.275	0.025	1.068	0.101	0.945	0.078	2.955	0.250	0	0
	MOO-N2	0.194	0.011	1.868	0.126	0.028	0	0	0	1.038	0.048	0.734	0.025	1.307	0.014	0.844	0.010	0	0
	MOO-N3	0.359	0.026	3.748	0.258	0.141	0.025	0	0	2.389	0.860	0.654	0.152	0.625	0.002	0.640	0.086	0	0
	MOO-S3	0.272	0.015	1.954	0.001	0	0	0	0	16.01	0.447	1.637	0.133	1.595	0.025	0.709	0.051	0	0
**Soil**	McG-N1-SF	0.044	0.008	0.198	0.024	0.021	0.002	0.405	0.120	0.630	0.025	0.884	0.054	0.522	0.035	0	0	0.056	0.025
	McG-N2-SF	0.112	0.011	0.357	0.015	0.014	0.002	0.231	0.003	1.652	0.001	0.812	0.021	2.709	0.111	0	0	0.080	0.025
	McG-N3-SF	0.101	0.021	0.229	0.033	0	0	0.541	0.041	2.230	0.050	0.336	0.022	4.504	0.125	0	0	0.229	0.024
	McG-S2-SF	0.120	0.019	0.771	0.016	0	0	0	0	2.353	0.014	1.055	0.310	3.018	0.010	0	0	29.245	0.257
	McG-S3-SF	0.101	0.022	1.003	0.251	0.014	0.003	0.275	0.022	2.802	0.125	0.928	0.028	4.424	0.095	1.128	0.250	0	0
	MOO-N1-SF	0.117	0.023	0.382	0.048	0	0	0.095	0.001	2.855	0.115	1.013	0.015	4.918	0.083	0.318	0.036	0	0
	MOO-N2-SF	0.107	0.022	0.421	0.098	0.010	0.005	0.353	0.025	2.060	0.068	0.586	0.123	1.724	0.012	0.581	0.025	0	0
	MOO-N3-SF	0.147	0.011	0.411	0.015	0.006	0.002	0.021	0.003	2.300	0.074	1.174	0.128	3.749	0.099	0.896	0.350	0	0
	MOO-S1-SF	0.122	0.025	0.481	0.003	0.015	0.001	0.194	0.004	4.306	0.115	0.986	0.025	3.176	0.085	1.092	0.005	0	0
	MOO-S3-SF	0.130	0.011	0.513	0.014	0	0	0.166	0.015	3.185	0.569	0.758	0.014	3.354	0.358	0.505	0	0	0
	McG-N1-10	0.059	0.012	0.664	0.005	0.038	0.003	0.423	0.012	0.833	0.004	0.852	0.144	0.733	0.012	0	0	0	0
	McG-N2-10	0.088	0.005	0.379	0.015	0	0	0.272	0.157	0.847	0.025	0.768	0.102	2.535	0.015	0	0	0.099	0.012
	McG-N3-10	0.092	0.008	0.671	0.122	0.018	0	0.223	0.110	3.115	0.224	0.681	0	1.862	0.250	0	0	0.226	0.005

	McG-S2-10	0.102	0.009	1.435	0.027	0	0	0.316	0.001	1.574	0.220	1.019	0.035	1.679	0.113	0	0	0.136	0.082
	McG-S3-10	0.098	0.011	1.444	0.147	0.001	0	0.447	0.005	1.627	0.221	0.753	0.021	5.100	0.052	0.951	0.250	0	0
	MOO-N1-10	0.249	0.025	1.017	0.004	0	0	0.676	0.001	1.951	0.014	0.543	0.001	6.917	0.047	3.154	0.025	0	0
	MOO-N2-10	0.060	0.004	0.515	0.008	0	0	0.873	0.250	1.384	0.075	1.010	0.036	2.258	0.157	0.266	0.001	0	0
	MOO-N3-10	0.243	0.031	1.201	0.124	0.002	0	0.741	0.001	2.187	0.119	0.392	0.001	7.262	0.458	3.210	0.875	0	0
	MOO-S1-10	0.118	0.009	0.523	0.036	0	0	0.344	0.026	2.671	0.226	0.767	0.025	5.398	0.112	0.977	0.098	0	0
	MOO-S3-10	0.116	0.011	0.386	0.017	0	0	0.544	0.119	2.093	0.014	0.493	0.112	3.415	0.321	0.705	0.066	0	0
**Loose Rock**	McG-N1	0.034	0.012	0.287	0	0.053	0	0	0	0.631	0.001	0.146	0.013	0.061	0.034	0	0	0	0
	McG-N3	0.086	0.006	6.046	0.684	0.051	0.027	0	0	2.515	0.913	0.191	0.038	0.128	0.015	0.907	0.446	0	0
	McG-S2	0.055	0.018	0.499	0.025	0.043	0.015	0	0	1.007	0.179	0.161	0.024	0.095	0.009	0	0	0	0
	McG-S3	0.090	0.036	0.468	0.150	0.149	0.032	0	0	0.908	0.114	0.385	0.090	0.146	0.028	0.188	0.040	0	0
	MOO-N1	0.065	0.015	0.269	0.017	0.036	0.004	0	0	0.923	0.053	0.215	0.024	0.117	0.013	0.731	0.103	0	0
	MOO-N2	0.063	0.008	0.330	0.033	0.058	0.014	0	0	1.037	0.040	0.164	0.020	0.105	0.003	0.481	0.099	0	0
	MOO-N3	0.092	0.047	0.868	0.074	0.066	0.003	0	0	1.316	0.051	0.530	0.100	0.122	0.038	1.078	0.328	0	0
	MOO-S1	0.081	0.029	4.060	1.891	0.038	0.022	0	0	1.523	0.579	0.233	0.094	0.138	0.035	0.430	0	0	0
	MOO-S3	0.159	0.031	0.481	0.181	0.042	0.014	0	0	3.986	1.464	0.271	0.183	0.078	0.045	0	0	0	0

SUPPORTING INFORMATION Fig.S6

Microbial community data analysis

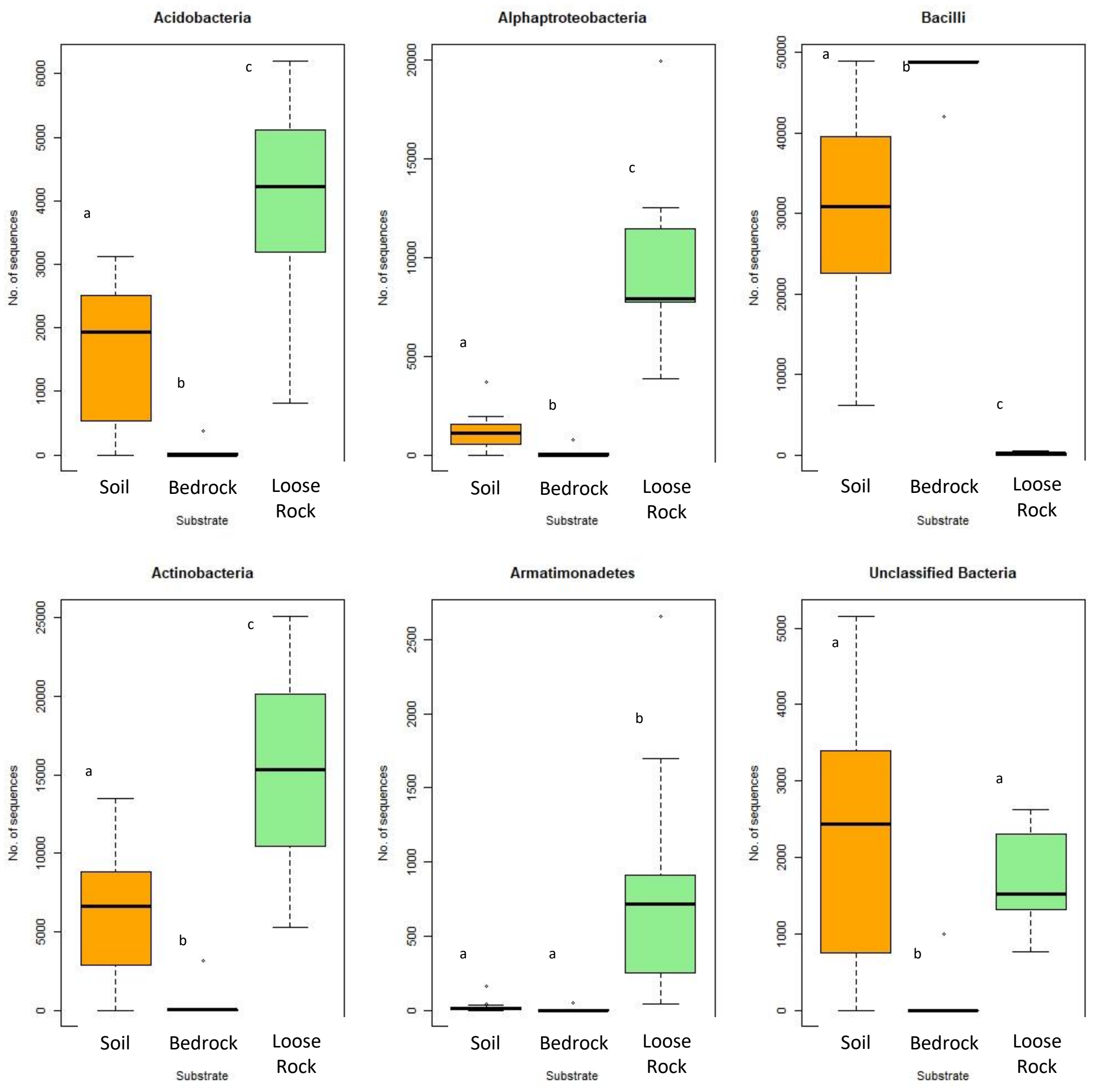

Boxplots presenting the significant differences for microbial classes detected by Illumina MiSeq sequencing at each of the analysed substrates. Different letters depict significant differences (*p* < 0.05) among the substrates.

SUPPORTING INFORMATION Fig.S6

Microbial community data analysis (cont.)

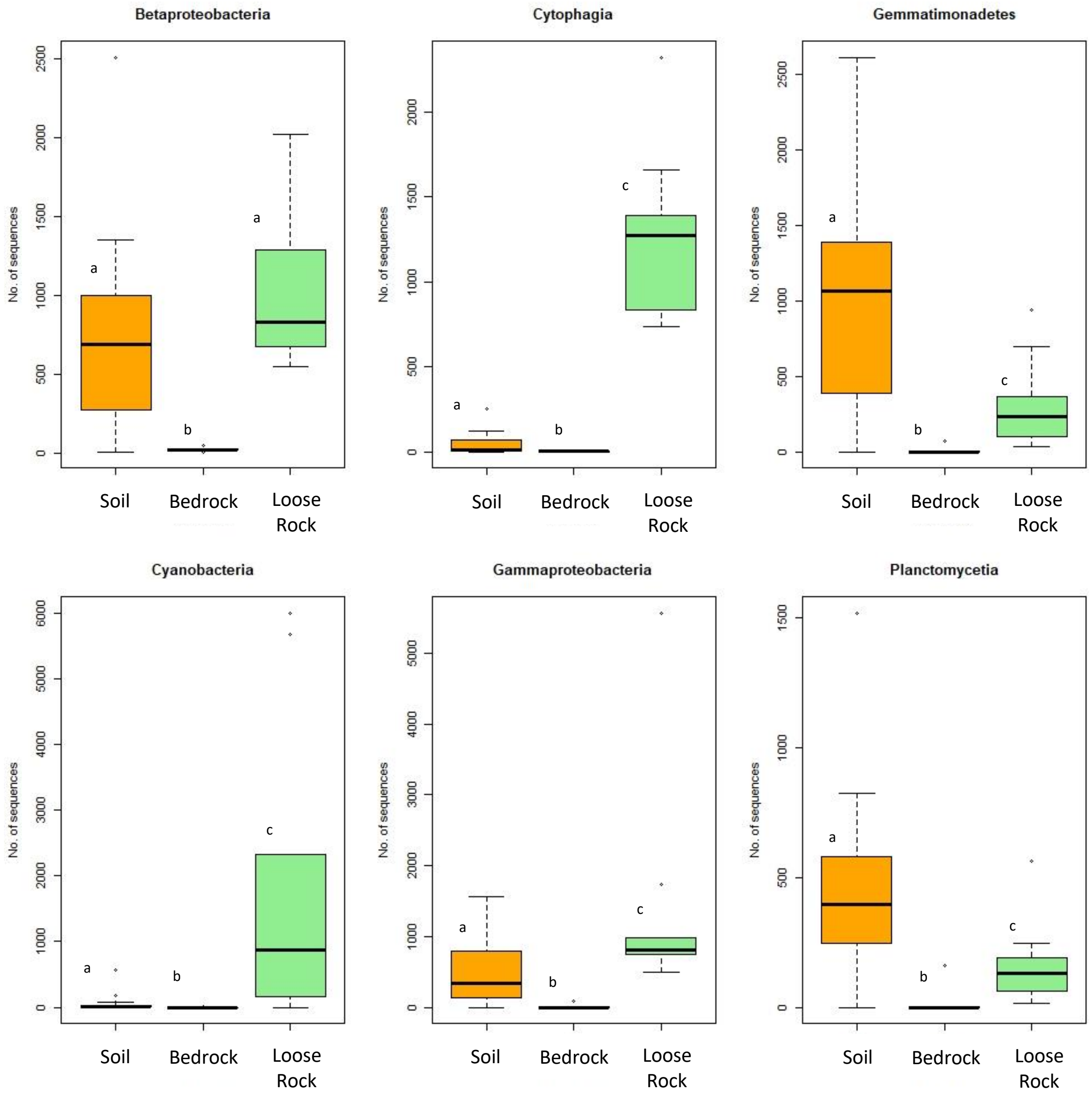

Boxplots presenting the significant differences for microbial classes detected by Illumina MiSeq sequencing at each of the analysed substrates. Different letters depict significant differences ($p < 0.05$) among the substrates

SUPPORTING INFORMATION Fig.S6

Microbial community data analysis (cont.)

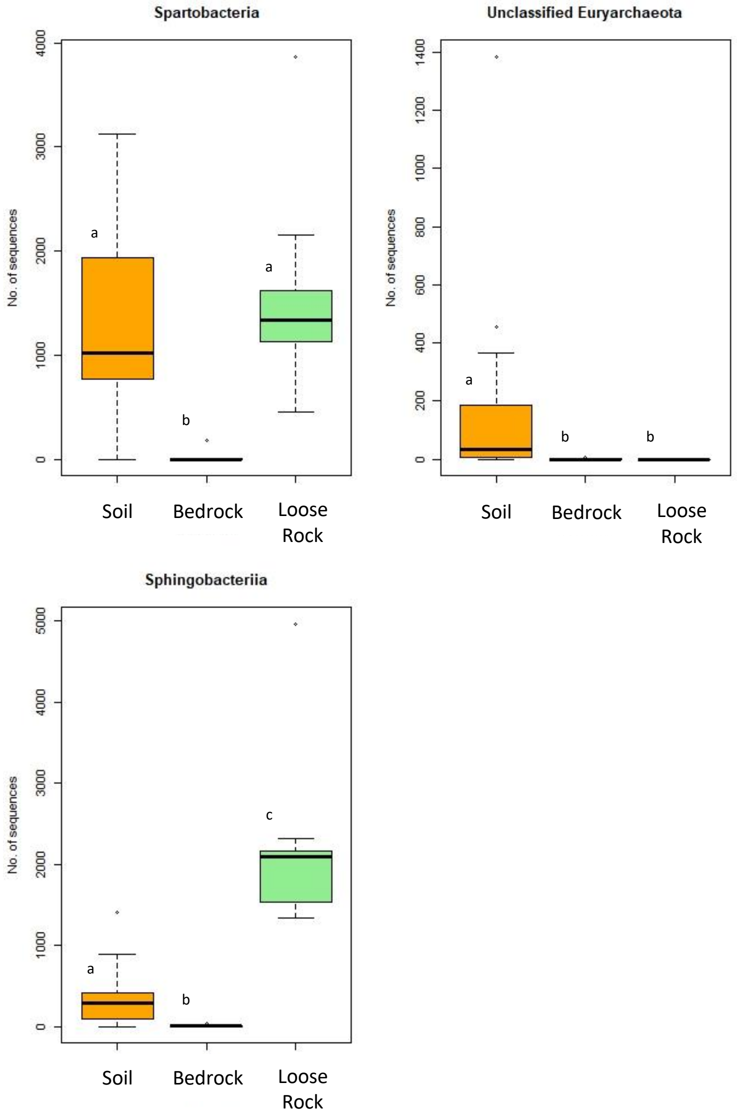

Boxplots presenting the significant differences for microbial classes detected by Illumina MiSeq sequencing at each of the analysed substrates. Different letters depict significant differences ($p < 0.05$) among the substrates

SUPPORTING INFORMATION Fig.S7

Venn diagram of shared microbial genera among substrates

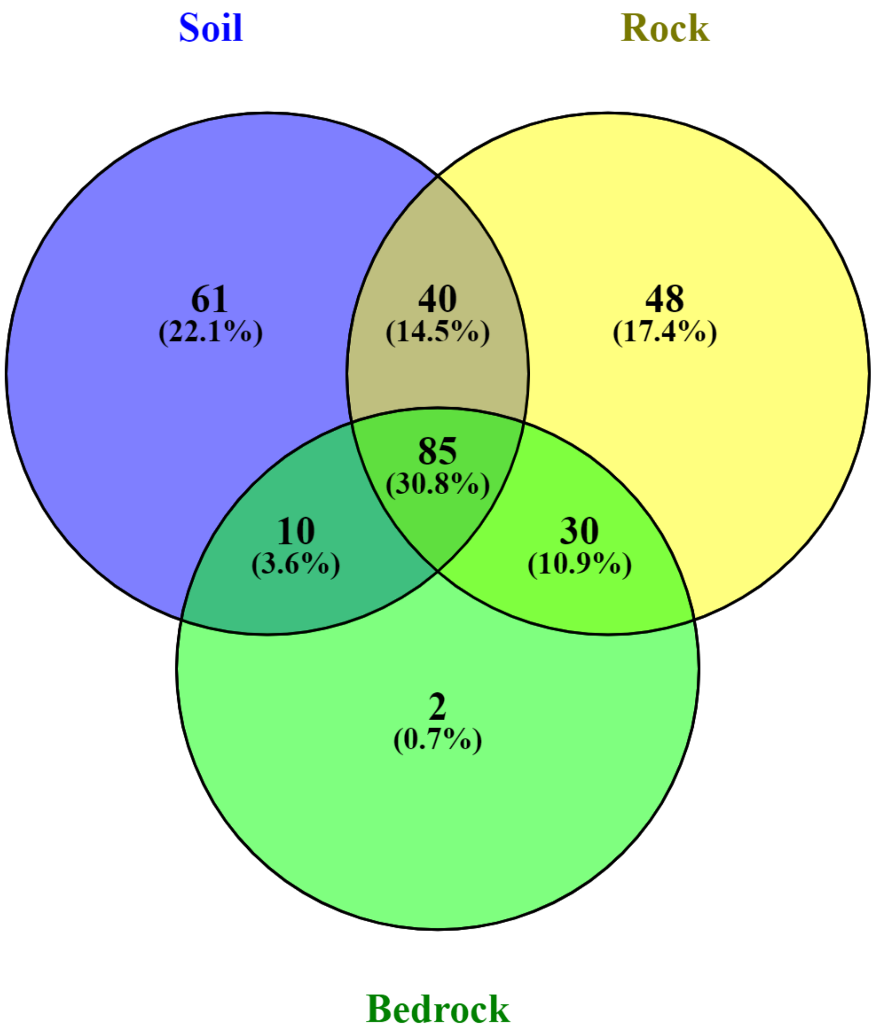

Venn diagram showing the number of unique (*i.e.* only found at one depth) and shared microbial genera detected among different substrates

SUPPORTING INFORMATION Fig.S8

Total organic carbon (TOC) and total nitrogen (TN) contents and bulk stable isotopic ratios (δ ^{13}C and δ ^{15}N)

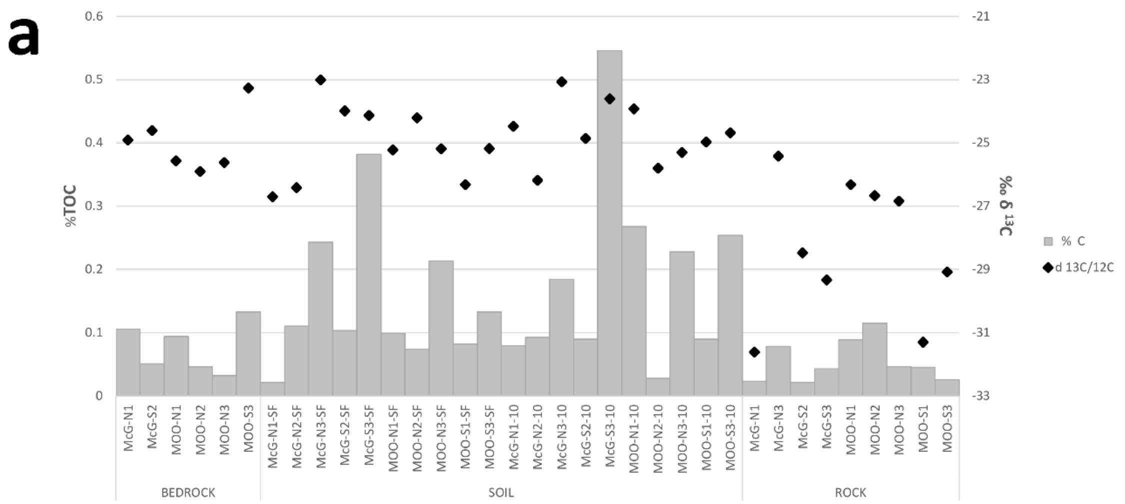

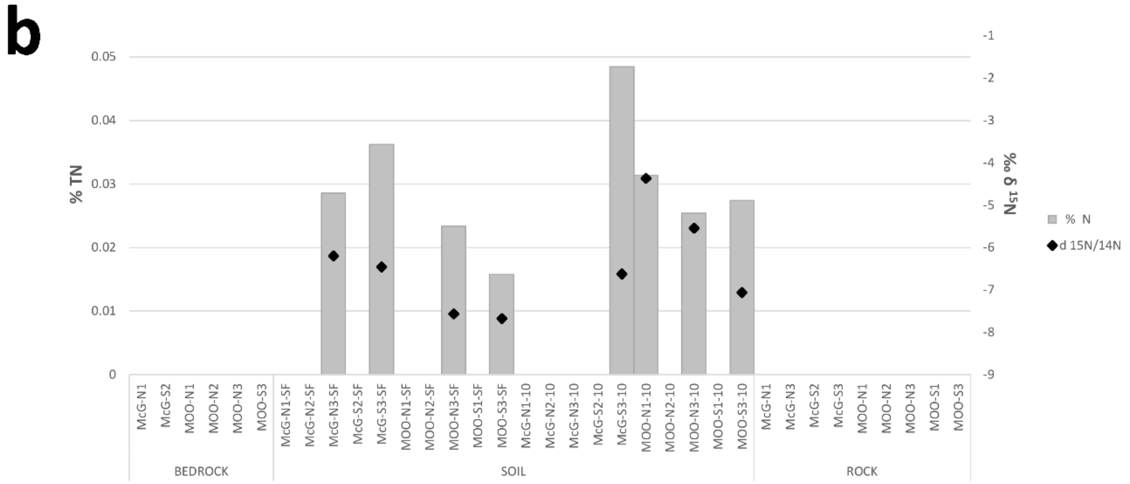

Carbon and nitrogen composition in the three substrates. (a) Total organic carbon (TOC, %), content (bars) and bulk $\delta^{13}C$ ratio (black diamonds). (b) Total nitrogen (TN, %) contents (bars) and bulk $\delta^{15}N$ ratio (black diamonds). Note that both Y-axes scale is different in the two plots.
